# Myeloid cell expansion propels right ventricular dysfunction in HFpEF via sterile inflammation

**DOI:** 10.1161/CIRCULATIONAHA.125.078476

**Published:** 2026-08-18

**Authors:** Lara Jaeschke, Ceren Koçana, Alexandra Maria Chitroceanu, Annika Winkler, Hannah Kleitke, Pauline Fahjen, Justus Kamp, David Faidel, Erik Asmus, Martin Meiser, Efstathios G Stamatiades, Karolin W. Hublitz, Veronika Zach, Lucie Kretzler, Daniela Zurkan, Paul-Lennard Perret, Leonardo A. von der Ohe, Kai Beckschulte, Szandor Simmons, Jonathan L. Gillan, Kristina Franz, Lina Alasfar, Virginia S. Hahn, Kavita Sharma, Edwardo Reynolds, Gabriele G. Schiattarella, Sophie van Linthout, Burkert Pieske, Niklas Beyhoff, Christiane Ott, Niklas Hegemann, Philipp Mertins, Frank Edelmann, Wolfgang M. Kuebler, Jana Grune

**Affiliations:** 1 Department of Cardiothoracic and Vascular Surgery, Deutsches Herzzentrum der Charité (DHZC), Berlin, Germany; 2 Charité-Universitätsmedizin Berlin, corporate member of Freie Universität Berlin and Humboldt-Universität zu Berlin, Berlin, Germany; 3 Institute of Physiology, Charité-Universitätsmedizin Berlin, corporate member of Freie Universität Berlin and Humboldt-Universität zu Berlin, Berlin, Germany; 4 DZHK (German Centre for Cardiovascular Research), Partner Site Berlin, Berlin, Germany; 5 Department of Cardiology, Angiology and Intensive Care Medicine, Campus Virchow Klinikum, Deutsches Herzzentrum der Charité, Berlin, Germany; 6 Proteomics Platform, Max-Delbrück-Center for Molecular Medicine, Berlin, Germany; 7 Junior Research Group Cardiac Aging and Nutrition German Institute of Human Nutrition Potsdam-Rehbruecke, Nuthetal, Germany; 8 Institute of Microbiology, Infectious Diseases and Immunology (I-MIDI), Berlin, Germany; 9 Department of Internal Medicine and Cardiology, Campus Virchow Klinikum, Charité - Universitätsmedizin Berlin, Berlin, Germany; 10 Berlin Institute of Health at Charité-Universitätsmedizin Berlin, BIH Center for Regenerative Therapies (BCRT), Berlin, Germany; 11 Experimental and Clinical Research Center, Charité-Universitätsmedizin Berlin and Max Delbrück Center for Molecular Medicine, Berlin, Germany; 12 Max Delbrück Center, Berlin, Germany; 13 Department of Cardiology, Campus Virchow Klinikum, Charité-Universitätsmedizin Berlin, corporate member of Freie Universität Berlin and Humboldt Universität zu Berlin, Berlin, Germany; 14 Department of Pediatric Hematology, Oncology and SCT, Campus Virchow Klinikum, Charité-Universitätsmedizin Berlin, corporate member of Freie Universität Berlin and Humboldt-Universität zu Berlin, Berlin, Germany; 15 Division of Cardiology, Johns Hopkins University School of Medicine, Baltimore, MD, USA; 16 Deutsches Herzzentrum der Charité, Department of Cardiology, Angiology and Intensive Care Medicine, Max Rubner Center for Cardiovascular Metabolic Renal Research (MRC), Charité-Universitätsmedizin Berlin, Germany, Berlin, Germany; 17 Translational Approaches in Heart Failure and Cardiometabolic Disease, Max Delbrück Center for Molecular Medicine in the Helmholtz Association, Berlin, Germany; 18 Division of Cardiology, Department of Advanced Biomedical Sciences, Federico II University, Naples, Italy; 19 Friede Springer Cardiovascular Prevention Center at Charité – Universitätsmedizin Berlin, Germany; 20 Experimental and Clinical Research Center (ECRC), a Cooperation of Charité-Universitätsmedizin Berlin and Max Delbruck Center for Molecular Medicine (MDC); 21 DZL (German Centre for Lung Research), partner site Berlin, Berlin, Germany

**Keywords:** HFpEF, RV dysfunction, myeloid cell recruitment, macrophages, sterile inflammation

## Abstract

**Background:**

Leukocyte’s role in frequently occurring right ventricular dysfunction (RVD) secondary to HFpEF remains poorly defined, partially due to the lack of suitable small animal models. Here, we followed a translational research approach by establishing a murine HFpEF model developing manifest RVD and analyzed human HFpEF cohorts to study the mechanistic link between leukocytes and RVD in HFpEF.

**Methods:**

<20-week-old (young) and >80-week-old (aged) male and female *C57BL/6J* mice were divided into four experimental groups: i) chow, ii) HFpEF (N[ω]-nitro-l-arginine methyl ester (L-NAME), high-fat diet), iii) chronic hypoxia (10% O_2_) and iv) HFpEF and hypoxia (RV-HFpEF) to assess bi-ventricular function and myeloid cell dynamics. To test whether myeloid cells are causally involved in the development of RV remodeling in HFpEF, we treated RV-HFpEF mice with a colony stimulating factor 1 receptor inhibitor to deplete myeloid cells.

**Results:**

RV-HFpEF resulted in LV diastolic dysfunction indicated by increased E/E’ ratio, reduced global longitudinal peak strain, smaller end-diastolic diameters and increased isovolumetric relaxation time compared to chow. RV-HFpEF animals developed RV hypertrophy and RVD evident as increased Fulton’s index as well as elevated RV systolic pressures and reduced tricuspid annular plane systolic excursion, respectively. Total leukocyte, monocyte, and macrophage counts were elevated in RV tissue of RV-HFpEF compared to chow or LV tissue from young and aged RV-HFpEF animals. These data were confirmed by unbiased proteomic analyses of RV tissue from RV-HFpEF mice, demonstrating increased abundance of proteins involved in activation of the innate immune system, macrophage chemotaxis and leukocyte migration when compared to LV tissue or other experimental groups. Fate mapping experiments revealed that recruited monocyte-derived macrophages became the main source of total cardiac macrophages in RV tissue from RV-HFpEF mice. Depletion of myeloid cells was associated with lower RVSP profiles in RV-HFpEF mice compared to controls. In RV biopsies from HFpEF patients, we found increased expression of adhesion molecules, fibrotic markers, inflammatory transcripts and an association between RVD and CD68^+^ cells.

**Conclusion:**

We demonstrate that dysregulated myeloid cell dynamics are associated with, and directly contribute to, the pathogenesis of RVD-HFpEF in humans and mice.

## Introduction

Heart failure with preserved ejection fraction (HFpEF) is frequently associated with risk factors and comorbidities such as hypertension, older age, sedentary lifestyle, and obesity^1,2^. The synergism of hemodynamic and metabolic stress in concert with low-grade chronic inflammation is considered to contribute to disease manifestation and progression in the left ventricle (LV)^3,4^. Consistently, proinflammatory markers such as interleukin-6, tumor necrosis factor-alpha or high-sensitivity C-reactive protein have been established as predictors of clinical outcome in HFpEF patients, pointing toward a causal role of inflammation in HFpEF pathogenesis^5^. As of yet, macrophage expansion sourced by increased hematopoiesis and myeloid cell recruitment in HFpEF LV tissue, is widely accepted as a pathophysiological hallmark of diastolic dysfunction^6,7^. Of note, this HFpEF-associated dysregulation of systemic, sterile inflammation does not exclusively affect the LV, but also tissues and organs beyond the LV, such as the pulmonary microvascular system, the gut microbiome, and the kidneys^8–10^.

A subset of HFpEF patients develops secondary pulmonary hypertension (PH) and subsequent right ventricular dysfunction (RVD)^11–13^. Importantly, RVD is recognized as a negative prognostic factor in LV failure, independent of the underlying cause^14^. This ventricular interdependence has traditionally been attributed to increased RV afterload secondary to pulmonary congestion, leading to RV remodeling, dilation and dysfunction, resulting in shortness of breath, fatigue, and syncope in affected patients^15^. This paradigm has, however, been challenged by the fact that RVD is primarily related to LV dysfunction and not to systolic pulmonary artery pressure *per se*. Moreover, RVD has recently been observed in HFpEF patients in the absence of PH^16^, suggesting that chronic pulmonary artery pressure elevation alone is insufficient to explain RVD in HFpEF^17,18^. As such, mechanisms other than direct hemodynamic forces, such as interventricular signaling, metabolic effects, or inflammatory cues have been proposed to contribute to RVD in LV failure^19,20^, but little is known about the underlying pathophysiological mechanisms fueling the development of RVD in HFpEF.

Here, we investigate the role of myeloid cells in RVD in HFpEF, following the notion that the innate immune system and dysregulated sterile inflammation are key pathogenic drivers of HFpEF-associated RVD. Since small animal models fully recapitulating the clinical HFpEF syndrome are rare and often poorly phenotyped for RV function and dimensions^21^, we employed a novel mouse model of concomitant hypertensive, metabolic and hypoxic stress that phenocopies the development of RVD in HFpEF. In this model, myeloid cell accumulation, proliferation and recruitment were associated with, and directly contributed to, the pathogenesis of RVD in HFpEF mice via RV remodeling and tissue inflammation. Moreover, we show that HFpEF-RVs exhibit unique pathogenic responses compared to HFpEF-LVs, when exposed to the same systemic stressors, with HFpEF-RVs being particularly vulnerable to myeloid cell recruitment and extracellular matrix deposition. In the clinical scenario, we found that HFpEF patients with RV dilation had unique white blood cell signatures with a higher percentage of blood monocytes when compared to HFpEF patients with regular RV diameters. RV biopsies from HFpEF patients exhibited inflammatory and pro-fibrotic signatures and RV CD68^+^ cell counts associated with RV hemodynamics. Viewed together our data indicate a causal role for myeloid cells in HFpEF-associated RVD.

## Methods

Detailed methods are provided in the online supplement. The data that support the findings of this study are available from the corresponding author upon reasonable request. The mass spectrometry proteomics data have been deposited to the ProteomeXchange Consortium via the PRIDE^22^ partner repository with the dataset identifier PXD074772 (Table S1, S2).

### Human

#### HFpEF definition

HFpEF was defined in accordance with current ACC diagnostic guidelines^23^ as the presence of the following symptoms and signs: i) at a minimum New York Heart Association (NYHA) Class II, ii) a LVEF greater than 50%, and iii) objective evidence of cardiac structural abnormalities [left ventricular mass index (LVMI) > 95 g/m^2^ in females and > 115 g/m^2^ in males, relative wall thickness (RWT) > 0.42, left atrial volume index (LAVI) > 34 mL/m^2^] and/or functional abnormalities [E/e’ ratio at rest > 13, pulmonary artery systolic pressure (PASP) > 35 mmHg] indicative of LV diastolic dysfunction or elevated left ventricular filling pressures, and iv) elevated levels of natriuretic peptides [N-terminal pro-B-type natriuretic peptide (NT-proBNP) > 125 pg/mL]. Non-participants included patients with NTproBNP < 125 or NYHA class I.

#### CRC1470 cohort

HFpEF patients were prospectively enrolled in the clinical cohort of the Collaborative Research Center 1470 (CRC1470), a consortium funded by the German Research Foundation (DFG: Deutsche Forschungsgemeinschaft). The study was conducted in accordance with the Declaration of Helsinki and approved by the Charité – Universitätsmedizin Berlin (Berlin, Germany) Ethics Committee (ethical approval EA1/224/21). All participants were recruited at a single-center, at Charité – Universitätsmedizin Berlin, were over 18 years old and provided written informed consent. Patients were excluded if they had a life expectancy of less than one year, a history of heart failure with mid-range or reduced ejection fraction, acute coronary syndrome within the last 30 days, cardiac surgery within the past three months, or end-stage renal disease with or without hemodialysis. Additional criteria encompassed severe valvular heart disease, hypertrophic cardiomyopathy, amyloidosis, congenital heart diseases, sarcoidosis, and constrictive pericarditis. Patients with extracardiac conditions that could explain their symptoms, such as chronic obstructive pulmonary disease (COPD) GOLD stage > 2, primary pulmonary hypertension, or moderate to severe anemia (hemoglobin < 10 g/dL in males and < 9.5 g/dL in females) were also excluded. All patients underwent a comprehensive clinical evaluation, which included a two-dimensional echocardiography.

#### Bulk RNA sequencing and CD68^+^ quantification in human HFpEF biopsies

All data were pulled from a previously published data repository^24^. In brief, human HFpEF samples were obtained from patients who underwent right heart catheterization with endomyocardial biopsy sampling of the RV septum at Johns Hopkins University. HFpEF diagnosis was based on consensus criteria, as described previously^24^. Control RV septal tissue came from unused donor hearts from the University of Pennsylvania. Quantitative analysis of CD68^+^ cell counts using immunohistochemistry was performed using HALO (Area Quantification FL algorithm, Indica Lab, Albuquerque, NM)^24^. Differential gene expression was assessed using DESeq2 as previously described^24^ with removal of the filter for low expression. The normalized expression of selected genes was graphically illustrated.

### Mice

All animal studies performed are conform to Directive 2010/63/EU of the European Parliament, the guidelines of the German Law on the Protection of Animals and the Guide for the Care and Use of Laboratory Animals published by the US National Institutes of Health (NIH Publication No. 85-23, revised 1985). The experimental protocols were reviewed and approved by the local authorities (Landesamt für Gesundheit und Soziales, Berlin, Germany; protocol no. G0008/22 and G0025/24) and performed in agreement with the Animal Research: Reporting of In Vivo Experiments (ARRIVE) guidelines. Wildtype mice were purchased from Janvier-Labs. *Cx3cr1*^*CreER/+*^*R26*^*tdTomato/+*^ mice were kindly provided by Efstathios Stamatiades (Charité Berlin) for fate-mapping experiments and were generated by crossing *Cx3cr1*^*CreER/+*^ with *R26*^*tdTomato/tdTomato*^ mice^25,26^. Aged wildtype mice were kindly provided by Christiane Ott (German Institute of Human Nutrition Potsdam-Rehbruecke).

#### RVD induction in HFpEF mice

Mice were housed for at least 1 week to acclimatize to laboratory conditions before starting experimental procedures. We housed two to four mice per cage, divided by sex and treatment, kept in a 12-h:12-h light/dark cycle with free access to food and water in individually-ventilated cages and specific pathogen-free rooms.

<20-week-old male and female *C57BL/6J* mice were divided into four experimental groups via block randomization: i) naive mice receiving standard diet (chow, 9% fat, Ssniff) and regular drinking water, serving as controls, ii) mice receiving a rodent diet with 60% fat (high-fat diet, Ssniff) and Nω-nitro-L-arginine methylester hydrochloride (L-NAME, 0.5 g/L, N5751-10G, Sigma-Aldrich) diluted in drinking water for twelve weeks to induce HFpEF, iii) mice receiving high-fat diet, L-NAME diluted in drinking water with additional exposure to chronic hypoxia (10% O_2,_ RV-HFpEF) for two weeks starting from week 10 to induce RVD in HFpEF, iv) chow mice exposed to hypoxia for two weeks. Mice cohorts were age- and sex-matched and were run in parallel with all other experimental groups. For aging experiments, we used >80-week-old male and female *C57BL/6J* mice undergoing the RV-HFpEF model.

Study protocols were not registered beforehand. All outcome measurements were randomized to prevent systematic bias. Animals from different groups were distributed across multiple cages to avoid batch effects. Experimenters were aware of group allocation at the conduct of the experiment and outcome assessment, but not at data analysis.

## Statistics

Statistical analyses for human cohorts and proteomics were performed using R or SPSS. For two-group comparisons, we used Wilcoxon rank sum test. For categorical variables, we used Pearson Chi-squared test or Fisher’s exact test. Correlations between CD68^+^ cell counts and clinical data were assessed using linear correlation analyses using Pearson’s correlation coefficient r or the coefficient of determination R2. Shown is the 95% confidence interval. Proteomic spectra were processed with Spectronaut, and quantified protein groups were filtered for completeness and quality before normalization and imputation of missing values. Differential gene expression was assessed with limma using duplicate correlation, and pathway enrichment was evaluated by pre-ranked GSEA (fgsea package), using the Reactome or Gene ontology pathway database with Benjamini–Hochberg correction for multiple testing. The ranking was performed on the t-statistics for each comparison, with each comparison treated as a single input vector representing coordinated protein-level changes between conditions.

For mouse studies, statistical analyses were performed using GraphPad Prism 9 and power analysis for sample size calculation was done using G*Power version 3.1. Results are reported as box plots using the 25th (Q1), 50th (median or Q2) and 75th (Q3) percentiles and the interquartile range (IQR = Q3 − Q1), which covers the central 50% of the data. Sample sizes below 20 were assumed to be not normally distributed by default, as a sample size below 20 may not provide enough power to detect significant differences between the sample data and the normal distribution. Outliers were removed by applying the ROUT outlier test. For two-group comparison, datasets were analyzed by unpaired non-parametric two-tailed Mann-Whitney test. For multiple comparisons, we used non-parametric Kruskal-Wallis test followed by uncorrected Dunn’s test. To compare the mean difference between groups with two independent variables two-way ANOVA with Fisher’s least significant difference (LSD) was used. Nested two-sided t-test or nested one-way ANOVA followed by uncorrected Fisher’s LSD test was applied when analyzing technical replicates. Statistical significance was assumed at p < 0.05.

## Results

### HFpEF is associated with macrophage accumulation in RV tissue in mice

**Fig. 1:**
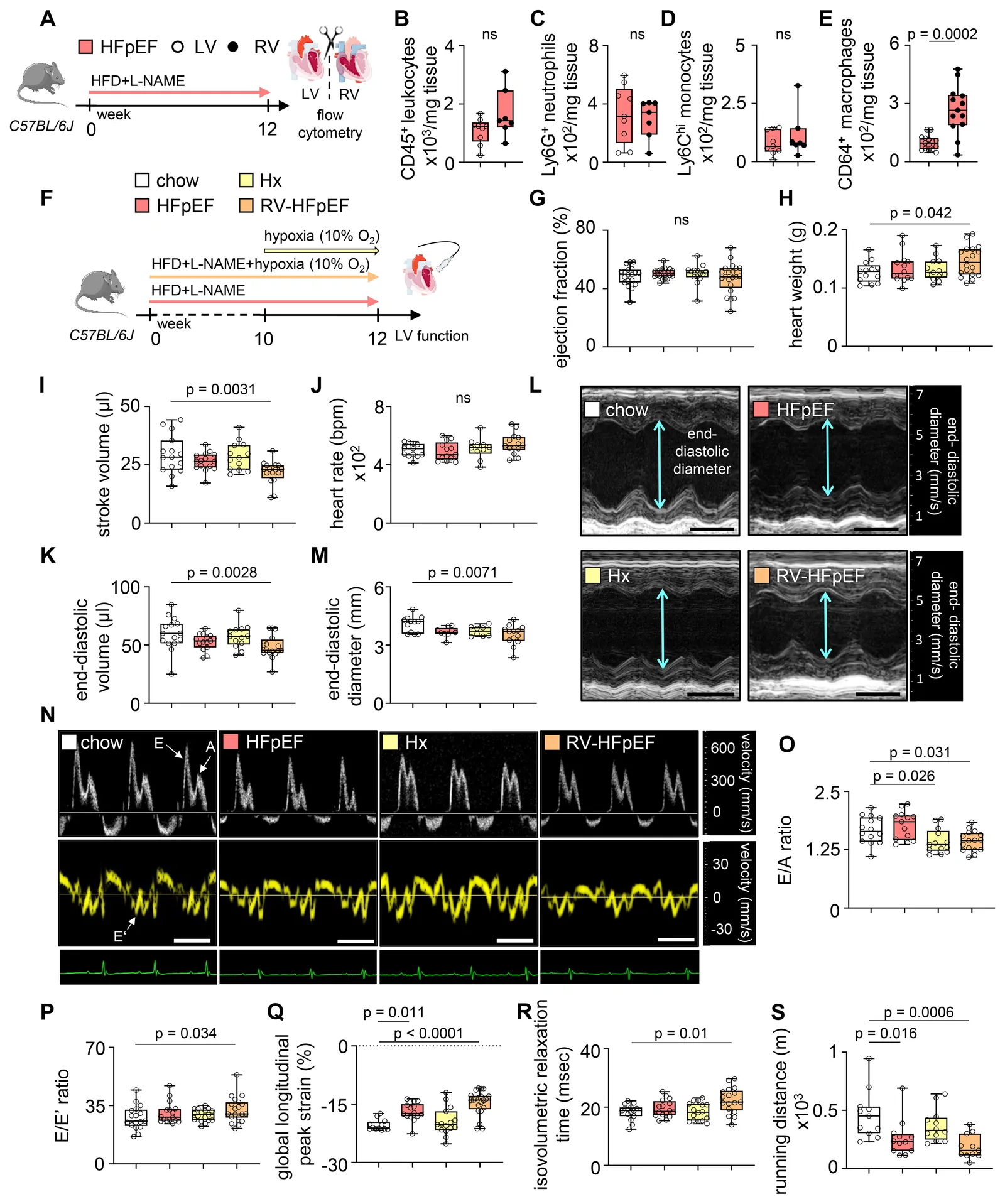
Chronic hypoxia exacerbates LV diastolic dysfunction in HFpEF. **A**, Experimental outline of HFpEF mice induced by treatment of *C57BL/6J* mice with L-NAME and 60% HFD. **B, C, D, E**, Flow cytometric analyses of LV and RV processed tissue samples from HFpEF mice: **B**, CD45^+^ leukocytes. **C**, Ly6G^+^ neutrophils. **D**, Ly6C^hi^ monocytes and **E**, CD64^+^ macrophages (n=7-15). **F**, Experimental outline of *C57BL/6J* mice treated with i) chow, ii) HFpEF (L-NAME, 60% HFD), iii) chronic hypoxia (Hx, 10% O_2_) or iv) HFpEF and hypoxia (RV-HFpEF). **G**, Echocardiographic assessment of ejection fraction in chow, HFpEF, Hx and RV-HFpEF (n=14-21) mice. **H**, Heart weight of chow, HFpEF, Hx and RV-HFpEF (n=13-18) mice. **I**, Echocardiographic assessment of stroke volume (n=13-16), **J**, Heart rate (n=10-14) and **K**, end-diastolic left ventricular volume in chow, HFpEF, Hx and RV-HFpEF (n=13-16**)** mice. **L**, Representative echocardiographic M-mode images of the LV in the parasternal long-axis view indicated with blue arrows. scale bar=1 ms. **M**, Analyses of end-diastolic LV diameter of chow, HFpEF, Hx and RV-HFpEF (n=8-13) mice. **N**, Representative echocardiographic pulsed-wave Doppler tracings to calculate the early (E) and late (A) wave peak velocities indicated with white arrows (top panel) and tissue Doppler tracings to calculate early filling velocity (E′) indicated with white arrow (middle panel). Scale bar=1 ms. **O**, E/A ratio (n=13-16), **P**, E/E’ ratio (n=17-19), **Q**, global longitudinal peak strain (n=12-21) and **R**, isovolumic relaxation time in chow, HFpEF, Hx and RV-HFpEF (n=15-17) mice. **S**, Running distance of chow, HFpEF, Hx and RV-HFpEF (n=11-12) mice in exercise exhaustion test. P values were calculated using a Mann–Whitney test (B-E) or a Kruskal-Wallis test followed by uncorrected Dunn’s multiple comparisons test(G-K, M, O-S). Data are presented as median with interquartile range (IQR). HFpEF, heart failure with preserved ejection fraction. LV, left ventricle. RV, right ventricle. HFD, high-fat diet. L-NAME, Nω-nitro-L-arginine methylester hydrochloride. ns, not significant.

To investigate myeloid cells in HFpEF, we employed the established two-hit mouse model for HFpEF (L-NAME, 60% high-fat diet) in *C57BL/6J* mice and dissected cardiac tissue from HFpEF mice in LV (including septum) and RV samples to assess leukocyte counts (Fig. 1A)^27^. Total CD45^+^ leukocyte counts trended higher in HFpEF-RVs compared to HFpEF-LVs, mainly due to expansion of cardiac CD64^+^ macrophages (Fig. 1B-E, Fig. S1A, B). To explore a possible association of macrophage expansion with RVD in mice undergoing the two-hit model, we assessed RV function, yet detected no signs of RV hypertrophy with systolic RV pressures in the physiological range (Fig. S1C).

### Oxygen deprivation fuels LV diastolic dysfunction

A substantial hurdle for studies on RVD is the lack of suitable small animal models^21^, as mice are generally protected from end-stage RV failure and develop only moderate PH and RV hypertrophy^28^. Hence, we designed a novel HFpEF mouse model associated with RVD by complementing the two-hit HFpEF model with an additional risk factor for PH and RVD development, namely exposure to chronic hypoxia, to induce manifest RVD in mice undergoing the two-hit HFpEF model. Of note, hypoxemia at rest and in particular during exercise is a common finding in HFpEF patients with a disease endotype of secondary PH and RVD^29–31^. Moreover, chronic hypoxia represents an important accelerator of proinflammatory and profibrotic processes, as Ly6C^lo^ monocytes were shown to sense hypoxia in maladaptive tissue remodeling^32^. Based on this evidence, we hypothesized that reduced oxygen levels in combination with concomitant metabolic and hypertensive stress would fuel RV remodeling and RVD development in the two-hit HFpEF model (henceforth referred to as the ‘RV-HFpEF’ model, Fig. 1F).

Exposure to chronic hypoxia (10% O_2_) for two weeks starting at week 10 after HFpEF induction in mice resulted in preserved EF, reduced stroke volumes, concentric LV hypertrophy, reduced LV end-diastolic diameters and LV volumes (Fig. 1G-M). RV-HFpEF mice presented with pronounced LV diastolic dysfunction indicated by decreased E/A ratios, increased E/E’ ratios, reduced global longitudinal peak strain, and increased isovolumetric relaxation time when compared to controls (Fig. 1N-R). Of note, exposure to chronic hypoxia alone induced signs of diastolic dysfunction independent of underlying HFpEF, confirming that oxygen depletion contributes to LV diastolic dysfunction independent of metabolic stress (Fig. 1N-R). Plasma NT-proBNP levels, gene expression of myosin heavy chain 7 (Myh7) and the natriuretic peptide B (Nppb), and hepatic triglycerides were increased in RV-HFpEF mice (Fig. S1D-F), suggestive of an aggravated HFpEF phenotype in RV-HFpEF mice. Exercise intolerance is a cardinal symptom of HFpEF patients, which we diagnosed in our animal model by shorter running distances of HFpEF mice when compared to normoxic controls in an established treadmill test (Fig 1S). Impaired LV function in RV-HFpEF mice relative to HFpEF mice was partially counteracted by increased hemoglobin content and hematocrit (Fig. S1G, H), resulting in an overall similar exercise performance. In sum, reduced oxygen availability exacerbates LV diastolic dysfunction.

### Chronic hypoxia induces RV remodeling and dysfunction in HFpEF mice

**Fig. 2:**
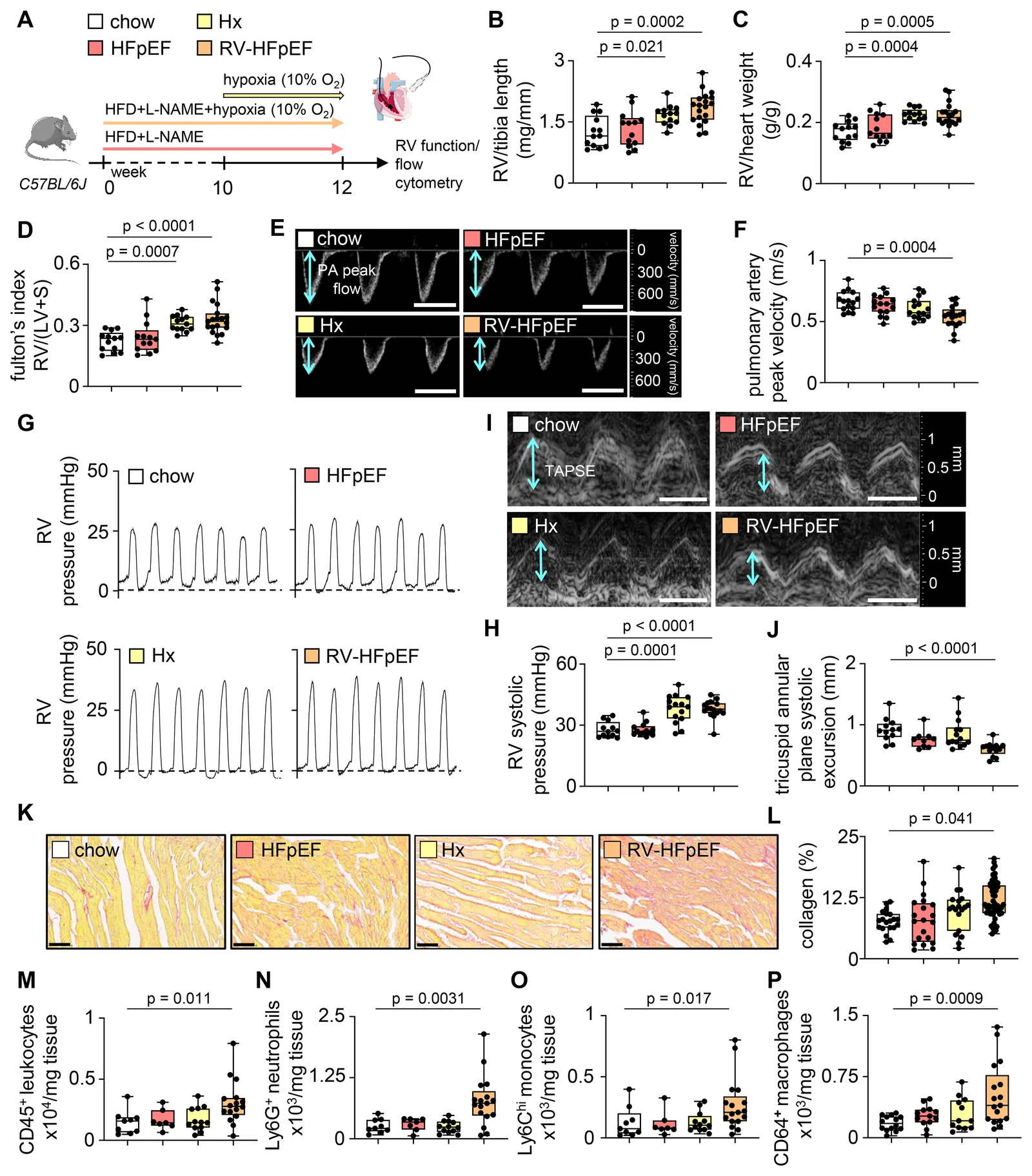
Combined hypoxia and HFpEF results in RVD. **A**, Experimental outline of *C57BL/6J* mice treated with i) chow, ii) HFpEF (L-NAME, 60% HFD), iii) chronic hypoxia (Hx, 10% O_2_) or iv) HFpEF and hypoxia (RV-HFpEF). **B**, RV weight-to-tibia length ratio (RV/tibia length, n=13-19), **C**, RV weight-to-heart weight ratio (RV/heart weight, n=12-19) and **D**, fulton’s index (RV mass relative to LV and septal mass (RV/(LV+S)) in chow in chow, HFpEF, Hx and RV-HFpEF (n=13-19) mice. **E**, Representative echocardiographic pulsed-wave Doppler tracings to calculate pulmonary artery peak velocity indicated with blue arrows. scale bar=1 ms. **F**, Quantitative analyses of pulmonary artery peak velocity profiles in chow, HFpEF, Hx and RV-HFpEF (n=15-17) mice. **G**, Representative RV systolic pressure profiles. **H**, Quantitative analyses of RV systolic pressures in chow, HFpEF, Hx and RV-HFpEF (n=12-16) mice. **I**, Representative echocardiographic M-mode images to calculate tricuspid annular plane systolic excursion (TAPSE) indicated with blue arrows. scale bar=1 ms. **J**, Quantitative analyses of TAPSE in chow, HFpEF, Hx and RV-HFpEF (n=9-14) mice. **K**, Representative images of picrosirius red staining of RV tissue sections. scale bar=50 µm. **L**, Quantitative analyses of collagen content in RV sections of chow, HFpEF, Hx and RV-HFpEF (n=20-55). Each dot represents a field of view. **M**, Flow cytometric quantification of RV CD45^+^ leukocytes, **N**, Ly6G^+^ neutrophils, **O**, Ly6C^hi^ monocytes and **P**, CD64^+^ macrophages of chow, HFpEF, Hx and RV-HFpEF (n=7-17) mice. P values were calculated using a Kruskal-Wallis test followed by uncorrected Dunn’s multiple comparisons test (B-D, F, H, J, M-P) or a nested one-way ANOVA followed by uncorrected Fisher’s least significant difference test (L). Data are presented as median with interquartile range (IQR). HFpEF, heart failure with preserved ejection fraction. LV, left ventricle. RV, right ventricle. HFD, high-fat diet. L-NAME, Nω-nitro-L-arginine methylester hydrochloride. TAPSE, Tricuspid Annular Plane Systolic Excursion.

RV phenotyping revealed RV hypertrophy in hypoxia-treated groups, independent of underlying HFpEF, evident as increased RV weights and Fulton’s index (Fig. 2A-D). At the functional level, we found decreased pulmonary artery peak velocities, elevated RV systolic pressures, and reduced echocardiographically-assessed tricuspid annular plane systolic excursions in RV-HFpEF mice, altogether indicating manifest RVD in RV-HFpEF mice (Fig. 2E-J).

Women represent a greater proportion of HFpEF patients when compared to HF with reduced EF (HFrEF) and outnumber men by a ratio of 2:1^33,34^. In contrast, female mice exposed to the two-hit HFpEF model were protected from LV diastolic dysfunction^35^. Differently from findings in the two-hit model, we did not observe sex differences in LV or RV outcomes in our novel RV-HFpEF mouse model (Fig. S2A-J), matching the clinical scenario where female sex is one of the strongest risk factors for pulmonary arterial hypertension (PAH) and associated RVD development^36^.

Next, we detected increased gene expression of pro-fibrotic markers and substantial collagen deposition in RV tissues from RV-HFpEF mice, but not in groups exposed to HFpEF or chronic hypoxia alone, indicating that a combination of concomitant metabolic, hypertensive and hypoxic stress is necessary to induce substantial RV remodeling (Fig. 2K, L, Fig. S2K). To test, whether myeloid cells contribute to RV remodeling in RV-HFpEF, we performed multi-color flow cytometry of RV tissues and found elevated CD45^+^ leukocyte, Ly6G^+^ neutrophil, Ly6C^hi^ monocyte, and resident CD64^+^ macrophage counts relative to controls (Fig. 2M-P). Of note, RV immune cell accumulation and inflammation occurred independent of sex in RV-HFpEF mice (Fig. S3A-G) and were not triggered by chronic hypoxia or metabolic stress alone,

but only became evident when combining metabolic stress, hypertension and chronic hypoxia (Fig. S4A-E), underscoring the relevance of concomitant systemic stressors for RVD development in HFpEF. In addition, RV-HFpEF reduced the percentage of resident macrophage subsets (Fig. S4F, G) and increased cardiac T-cell, B-cell and natural killer cell counts (Fig. S4H-K), indicating fundamental dysregulation of leukocyte dynamics in RVD associated to HFpEF.

### Dysregulated innate immune responses and extracellular matrix disorganization denote key pathophysiological features of RV tissue remodeling in HFpEF

**Fig. 3:**
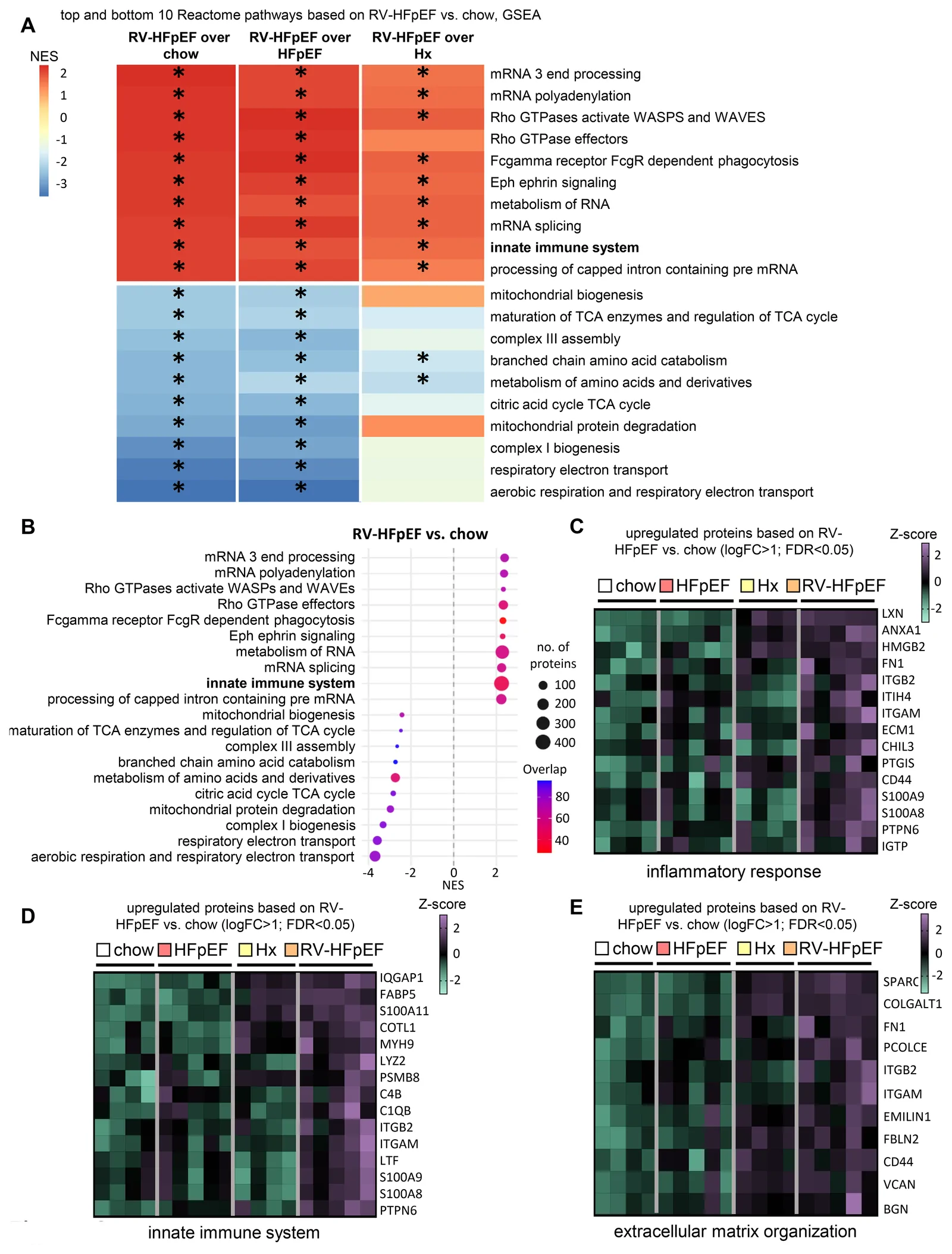
Innate immune system activation in RVD associated with experimental HFpEF. **A**, Heatmap showing the 10 most positively and 10 most negatively enriched Reactome pathways identified by pre-ranked GSEA for RV-HFpEF versus chow, together with the NES of these pathways in the other experimental comparisons (RV-HFpEF vs HFpEF and Hx). Pathway enrichment was calculated using proteins ranked according to moderated t-statistics derived from differential expression analyses for each comparison (red, positive enrichment; blue, negative enrichment). Asterisks indicate significantly enriched pathways (FDR < 0.05). The pathway marked in bold (“innate immune system”) confirms our initial findings from flow cytometry (leukocyte expansion in RV-HFpEF RVs). **B**, Dot plot showing the top 10 positively and top 10 negatively enriched Reactome pathways identified by GSEA for RV-HFpEF versus chow. A positive NES indicates enrichment of the respective pathway in RV-HFpEF relative to chow with a negative NES indicating the same for chow vs. RV-HFpEF. The size of each point represents the total number of proteins quantified in the MS workflow for the biological process and the colors of the points indicate the percentage that these proteins represent from the complete number of proteins annotated for the pathway in the Reactome database. NES were calculated with a weighted Kolmogorov–Smirnov-like running-sum statistic. **C, D, E**, Heatmaps of z-scored protein expression levels in RV tissue from chow, HFpEF, Hx, and RV-HFpEF mice (n = 4–5 per group). Shown are proteins that were significantly differentially expressed in the RV-HFpEF versus chow comparison (logFC > 1, FDR < 0.05), quantified in all samples without missing values, and annotated to the indicated Reactome or Gene Ontology Biological Process (GO) pathways (inflammatory response, innate immune system, and extracellular matrix organization). GSEA, Gene Set Enrichment Analysis. HFpEF, heart failure with preserved ejection fraction. Hx, hypoxia. NES, normalized enrichment score. RV, right ventricle.

To understand the molecular mechanisms involved in RV remodeling in HFpEF, we performed unbiased mass spectrometry-based proteomic profiling of bulk RV tissue from all experimental groups. Gene set enrichment analysis (GSEA) of Reactome pathways revealed upregulation of innate immune system in RV tissue from RV-HFpEF mice relative to other groups (Fig. 3A, B), confirming our initial findings from flow cytometry and histology. Importantly, these pathways remained significantly enriched when comparing RV-HFpEF proteomes with those from HFpEF-only or hypoxia-only mice, indicating that innate immune cell activation and extracellular matrix deposition are a consequence of the combined systemic stressors in RV-HFpEF. Enriched proteins from these pathways can be classified by function as follows: i) cell adhesion molecules such as integrin beta 2 (ITGB2), integrin alpha M (ITGAM), and cluster of differentiation 44 (CD44), ii) strong inflammatory mediators such as the alarmins S100 calcium-binding protein A8 (S100A8) and S100 calcium-binding protein A9 (S100A9), and iii) matricellular proteins such as versican (VCAN), secreted protein, acidic, rich in cysteine (SPARC) and fibulin-2 (FBLN2) (Fig. 3C-E). In addition, proteomic profiling revealed increased abundance of proteins associated with platelet activation and the adaptive immune system in RV-HFpEF mice (Tab. S1, S2), both pathophysiological features in clinical HFpEF^37–41^. Furthermore, RV tissues from RV-HFpEF mice presented with proteomic signatures indicative of impaired amino acid and mitochondrial metabolism, all of which represent pathophysiological hallmarks previously described in HFpEF myocardial tissue^42,43^.

### RV inflammation and myeloid cell expansion are age-related mechanisms of RVD-HFpEF

**Fig. 4:**
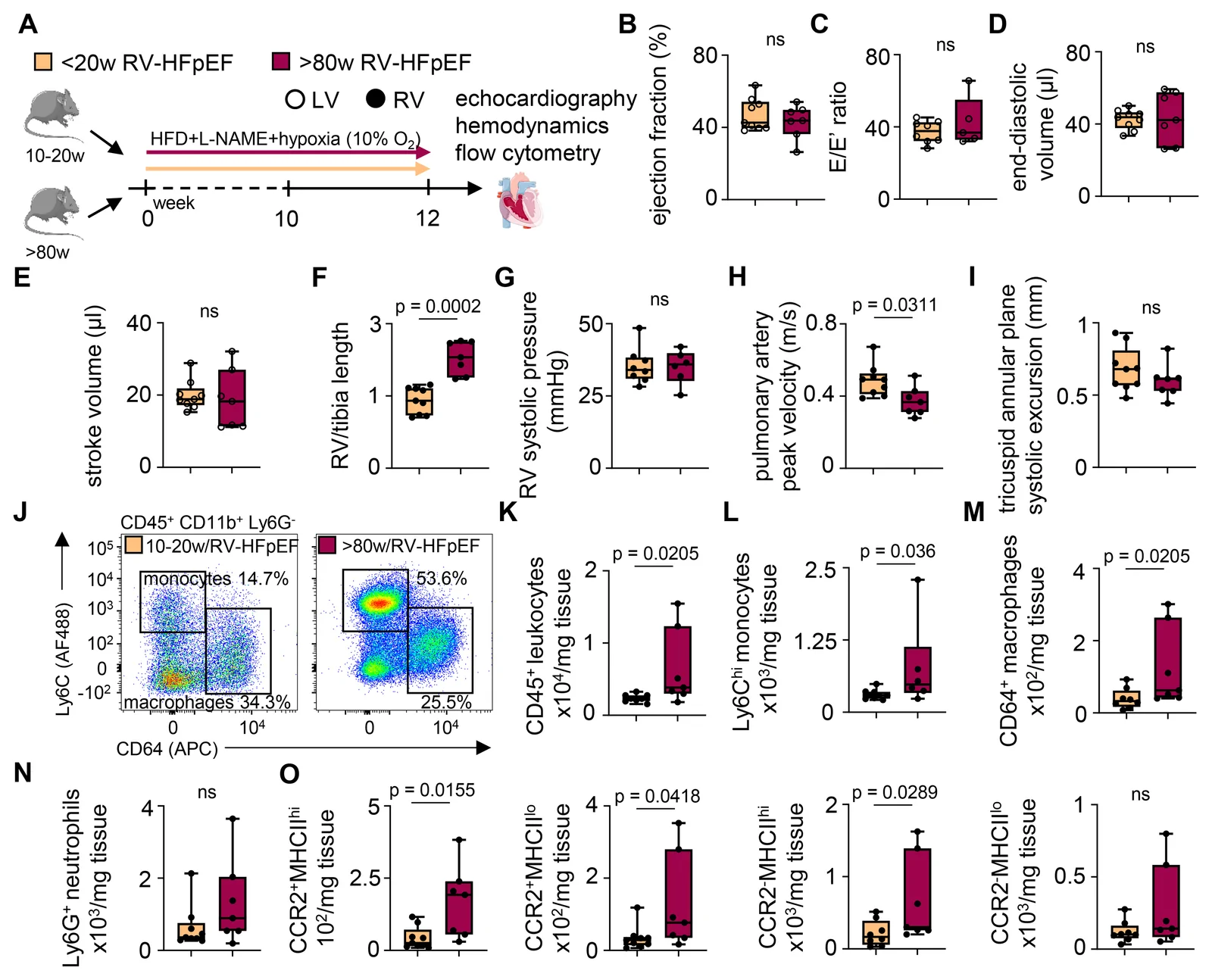
Myeloid cell expansion accelerates as a function of age in RV-HFpEF. **A**, Experimental outline of *C57BL/6J* mice treated with HFpEF (L-NAME, 60% HFD) and hypoxia (10% O_2_, RV-HFpEF) at <20 or >80 weeks of age. **B**, Echocardiographic assessment of LV ejection fraction (n=7-9), **C**, LV E/E’ ratio (n=5-8), **D**, LV end-diastolic volume (n=7-9) and **E**, LV stroke volume (n=7-9) in 10-20w RV-HFpEF and >80w RV-HFpEF mice. **F**, RV weight-to-tibia length ratio (RV/tibia length, n=7-9), **G**, RV systolic pressures (n=6-8), **H**, pulmonary artery peak velocity (n=7-9) and **I**, tricuspid annular plane systolic excursion (TAPSE, n=7-9) in <20w RV-HFpEF and >80w RV-HFpEF mice. **J**, Representative flow plots of RV myeloid cells in <20w RV-HFpEF and >80w RV-HFpEF mice. **K**, Flow cytometric quantification of RV CD45^+^ leukocytes (n=7-9), **L**, Ly6C^hi^ monocytes (n=7-9), **M**, CD64^+^ macrophages (n=7-8), **N**, Ly6G^+^ neutrophils (n=7-9) and **O**, CCR2^+^MHCII^hi^ (n=7-9), CCR2^+^MHCII^lo^ (n=7-9), CCR2^−^MHCII^hi^ (n=7-8) and CCR2^−^MHCII^lo^ (n=7-8) subsets in <20w RV-HFpEF and >80w RV-HFpEF mice. P values were calculated using a Mann–Whitney test (B-I, K-O). Data are presented as median with interquartile range (IQR). HFpEF, heart failure with preserved ejection fraction. LV, left ventricle. RV, right ventricle. HFD, high-fat diet. L-NAME, Nω-nitro-L-arginine methylester hydrochloride. ns, not significant.

The clinical endotype of HFpEF patients developing RVD is often older and presents with multiple comorbidities such as hypertension, atrial fibrillation or chronic obstructive pulmonary disease^44,45^. In light of the clinical presentation of RVD-HFpEF patients and to understand how increased biological age contributes to RV remodeling and dysfunction, we performed additional experiments with aged mice undergoing the RV-HFpEF model (Fig. 4A).

Initially, we screened RV myeloid cell profiles from aged animals (>80 weeks) and compared them to those from young mice (<20 weeks) to understand if increased biological age shapes RV myeloid cell dynamics in the absence of HFpEF (Fig. S5A). Increased biological age was *per se* associated with accumulation of Ly6C^hi^ monocyte, CD64^+^ macrophage and Ly6G^+^ neutrophil counts in RV tissue (Fig. S5B-E). Age-associated increased CD64^+^ macrophage expansion was mainly caused by higher CCR2^+^ macrophage counts, specifically the CCR2^+^MHCII^hi^ macrophage subset (Fig. S5F). Exposure of aged mice to RV-HFpEF resulted in comparable LV systolic and diastolic function in young and old mice, evident as similar ejection fractions, E/E’ ratios, end-diastolic volumes and stroke volumes (Fig. 4B-E). In contrast, RV weight was strongly increased in aged relative to young mice undergoing the RV-HFpEF model (Fig. 4F), suggestive of pronounced RV hypertrophy. RVD was partially aggravated in aged RV-HFpEF mice, demonstrated by lower pulmonary artery peak velocities at comparable RV systolic pressure profiles and TAPSE (Fig. 4G-I). At the cellular level, leukocyte expansion in RV tissues was more pronounced in aged than in young RV-HFpEF mice, and was mainly driven by Ly6C^hi^ monocytes and CD64^+^ macrophage accumulation (Fig. 4J-N). CCR2^+^ macrophage subsets were more abundant in RV tissue from aged RV-HFpEF mice when compared to young RV-HFpEF mice (Fig. 4O). These data identify RV myeloid cell expansion as a relevant pathophysiological feature of the aging process further accelerating RV remodeling and dysfunction in aged mice undergoing RV-HFpEF.

### HFpEF-RVs and HFpEF-LVs exhibit distinct ventricular remodeling responses when exposed to systemic stressors

**Fig. 5:**
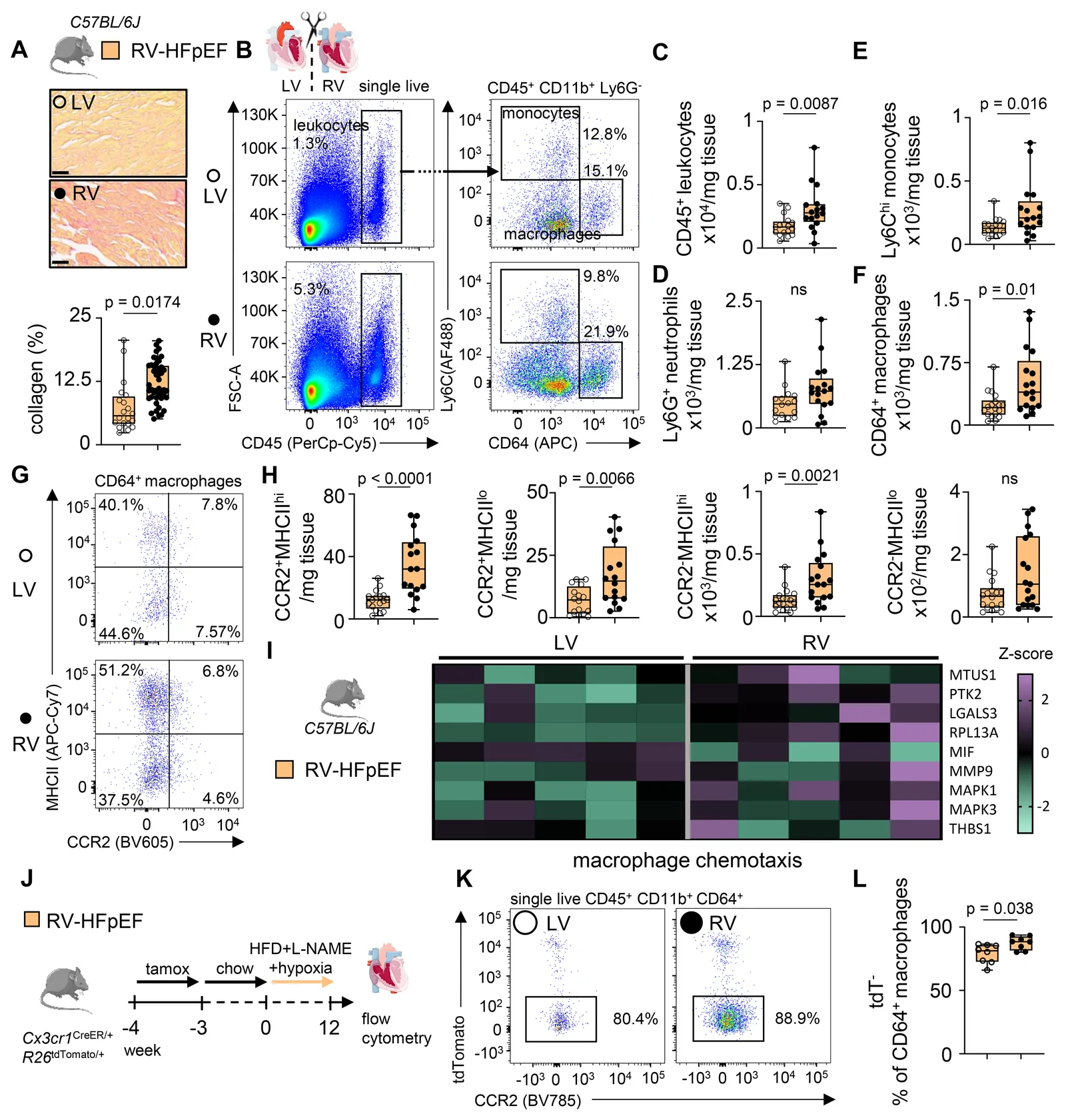
RV inflammation, myeloid cell recruitment and remodeling in RV-HFpEF mice. **A**, Representative images of picrosirius red staining. scale bar=50 µm (top). Quantitative analyses of collagen content in LV and RV (n=20-45) tissues from RV-HFpEF mice (bottom). Each dot represents a field of view. A nested t test was used. **B**, Representative flow plots of LV and RV leukocytes and myeloid cells in HFpEF mice exposed to hypoxia (10% O_2_, RV-HFpEF). **C**, Comparison of LV and RV CD45^+^ leukocytes, **D**, Ly6G^+^ neutrophils, **E**, Ly6C^hi^ monocytes and **F**, CD64^+^ macrophages in RV-HFpEF mice (n=16-18). **G**, Representative flow plots after gating for LV and RV macrophage subsets and **H**, corresponding quantification of CCR2^+^MHCII^hi^, CCR2^+^MHCII^lo^, CCR2^-^MHCII^hi^ and CCR2^-^MHCII^lo^ subsets in LV and RV (n=16-18) of RV-HFpEF mice. **I**, Heatmap of z-scored protein expression values in RV and LV tissue from RV-HFpEF mice (n=5). Shown are proteins annotated to the Gene Ontology term “macrophage chemotaxis” that have been detected in all input samples, RV and LV, from RV-HFpEF mice. **J**, Experimental outline of Fate-mapping in Cx3cr1^CreER/+^R26^tdTomato/+^ RV-HFpEF mice treated with tamoxifen-rich diet (tamox) for 1 week prior to a 3-week flushout followed by HFpEF (L-NAME, 60% HFD) and chronic hypoxia (Hx, 10% O_2_). **K**, Representative flow plots of LV and RV tdTomato negative (tdT^-^) macrophages in RV-HFpEF. **L**, Comparison of LV and RV (n=8) recruited tdT^-^ macrophages as a percentage of CD64^+^ macrophages. P values were calculated using a Mann– Whitney test (C-F, H, L). Data are presented as median with interquartile range (IQR). HFpEF, heart failure with preserved ejection fraction. LV, left ventricle. RV, right ventricle. HFD, high-fat diet. L-NAME, Nω-nitro-L-arginine methylester hydrochloride. Tamox, tamoxifen-rich diet. ns, not significant.

Cumulative evidence suggests that LV and RV fundamentally differ regarding their molecular, cellular and physiological adaptive response to adverse loading and systemic stressors^46^. To understand the role of myeloid cells in biventricular dysfunction due to systemic metabolic, hypertensive and hypoxic stress, we directly compared LV and RV tissue remodeling responses employing the RV-HFpEF model (Fig. 5). Interestingly, we found more pronounced collagen deposition in RVs accompanied by marked accumulation of total CD45^+^ leukocytes, Ly6C^hi^ monocytes and CD64^+^ macrophages when compared to LVs from the same animals (Fig. 5A-F). A similar pattern was evident for monocyte-derived CCR2^+^ macrophage subsets, which we found in higher numbers in RVs when compared to LVs, suggesting that the proportion of macrophages originally recruited from the blood pool of circulating monocytes is higher in RVs compared to LVs from RV-HFpEF animals, as the chemokine receptor CCR2 is preferentially expressed by recruited myeloid subsets^47^ (Fig. 5G, H). Accumulation of total macrophages and CCR2^+^ macrophage subsets in RV tissue was also detected in naïve aged mice and aged mice exposed to RV-HFpEF, expanding our initial findings to biological aging (Fig. S6A-K). The notion of ventricle-specific pathophysiological responses to systemic stressors was further supported by direct comparison of regulated pathways at the proteomic level (Fig. S7). In RV-HFpEF, proteins associated with innate immune system activation were significantly enriched in RV samples when compared to LV samples. Moreover, proteins involved in leukocyte migration and macrophage chemotaxis were enriched in RVs compared to LVs from RV-HFpEF mice (Fig 5I, Fig. S8), prompting us to exhibit experiments to decipher the origin and fate of RV macrophages in RVD-HFpEF.

*Monocyte recruitment is the major source of RV myeloid cell expansion in HFpEF-associated RVD*. Understanding origin and fate of immune cells has direct translational implications, as cells being causally involved in cardiac pathogenesis have been previously thought to be diagnostically inaccessible within the heart, but may be in reality quantifiable – and therefore targetable – in easily accessible peripheral blood samples. To explore the origin of macrophage expansion in RV-HFpEF ventricles, we first performed Ki67 staining on CD64^+^ macrophages in both ventricles from RV-HFpEF mice (Fig. S9A-B) to probe whether CD64^+^ macrophage expansion was attributable to proliferation of cardiac macrophages. In both ventricles, CD64^+^ macrophages presented with increased proliferation rates in response to RV-HFpEF, with no differences between LVs and RVs. As proliferation rates appeared comparable between the ventricles, we hypothesized that myeloid cell recruitment differed between the ventricles and represented the primary source of macrophage accumulation in RV tissue. To test this, we used *Cx3cr1*^*CreER/+*^*R26*^*tdTomato/+*^ mice for genetic fate mapping experiments in our novel RV-HFpEF model. In this reporter strain, *Cx3cr1*-expressing myeloid cells are efficiently labeled with tdTomato upon tamoxifen exposure (Fig. S9C-E). In RV-HFpEF mice, myeloid cell recruitment expanded so that over 80 % of ventricular macrophages derived from circulating monocytes, an increase compared to the steady state (data not shown). Of note, myeloid cell recruitment was significantly increased in RV tissue compared to LV tissue from the same RV-HFpEF animals (Fig. 5J-L). These results indicate that myeloid cell recruitment becomes the primary source of cardiac macrophages in HFpEF, which is exacerbated in RV tissue when compared to LV tissue.

### Myeloid cells propel RVD in HFpEF via sterile inflammation

**Fig. 6:**
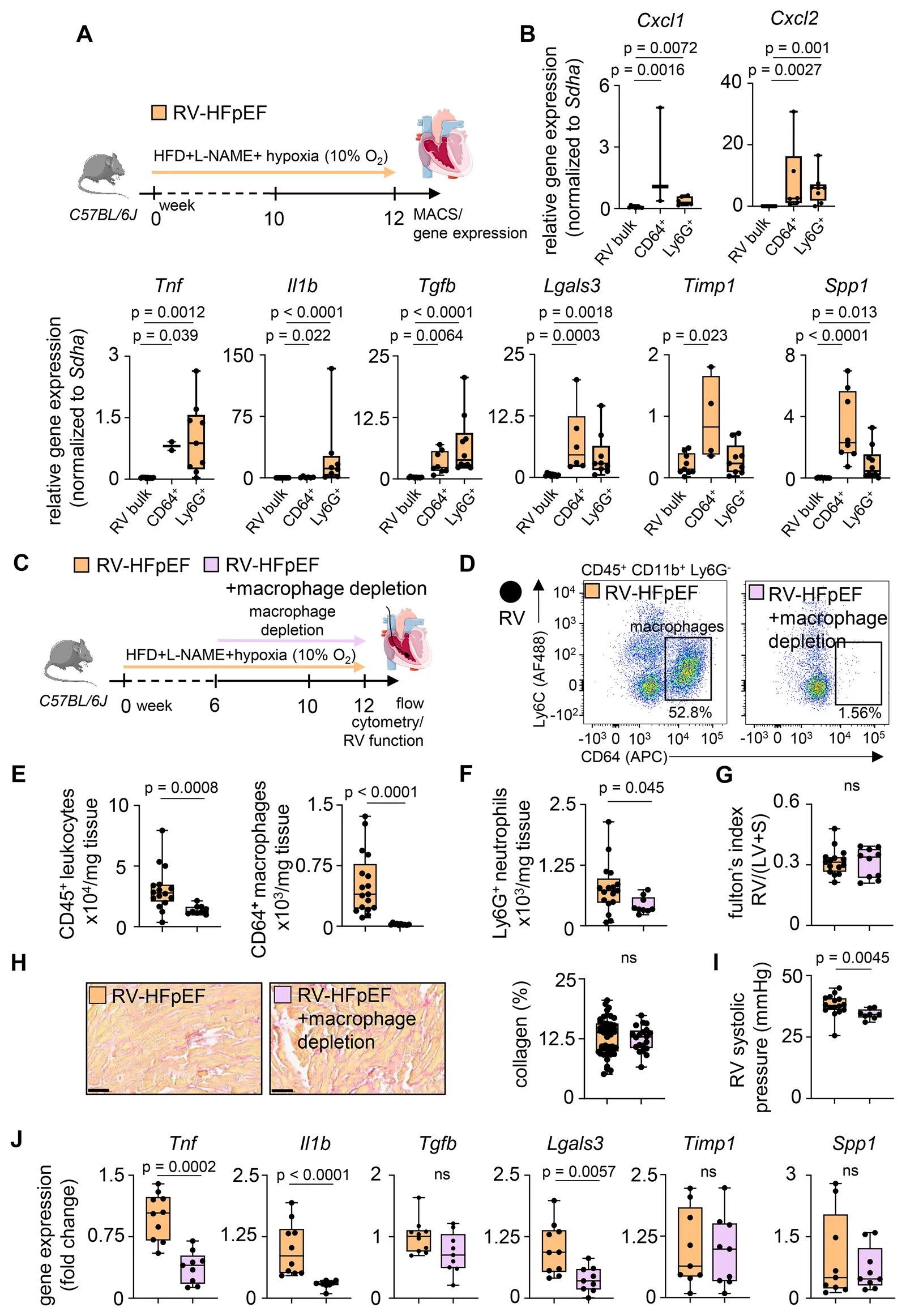
Macrophage depletion improves RV function in RV-HFpEF mice. **A**, Experimental outline. **B**, *Cxcl1, Cxcl2, Tnf*a, *Il1b, Tgfb, Lgals3, Timp1* and *Spp1* relative gene expression in RV bulk tissue, MACS sorted CD64^+^ macrophages and Ly6G^+^ neutrophils (n=2-10). Gene expression was normalized to *Sdha* using the 2^–ΔCt^ method. **C**, Experimental outline for RV-HFpEF mice treated with colony-stimulating factor 1 receptor (CSF1R) inhibitor (PLX5622) for the last 6 weeks of the experiment (RV-HFpEF+PLX5622). **D**, Representative flow plots showing macrophages from RV-HFpEF mice and RV-HFpEF+PLX5622 mice. **E**, Corresponding quantification of RV CD45^+^ leukocytes in RV-HFpEF and RV-HFpEF+PLX5622 (n=9-16) mice (left) and CD64^+^ macrophages in RV-HFpEF and RV-HFpEF+PLX5622 (n=10-17) mice (right). **F**, Quantification of Ly6G^+^ neutrophils in RV-HFpEF and RV-HFpEF+PLX5622 (n=10-17) mice. **G**, Fulton’s index (RV weight to LV + septum weight (RV/(LV+S))) from RV-HFpEF and RV-HFpEF+PLX5622 (n=10-15) mice. **H**, Representative images of Picrosirius red staining (left). scale bar=50 µm. Quantitative analyses of RV-HFpEF RVs and RV-HFpEF+PLX5622 (n=20-45) collagen content (right). Each dot represents a field of view. Nested t test was used. **I**, RV systolic pressure of RV-HFpEF and RV-HFpEF+PLX5622 (n=8-16) mice. **J**, Quantitative PCR of RV-HFpEF and RV-HFpEF+PLX5622 mice. *Tnf* (n=9-10), *Il1b* (n=8-10), *Tgfb* (n=9-10), *Lgals3* (n=9-10), *Timp1* (n=9) and *Spp1* expression in RV bulk tissue of RV-HFpEF and RV-HFpEF+PLX (n=9-10). Expression values were calculated relative to *Sdha* expression using the 2^–ΔΔCt^ method. P values were calculated using a Kruskal-Wallis test followed by uncorrected Dunn’s multiple comparisons test (B), or a Mann–Whitney test (E-J). Data are presented as median with interquartile range (IQR). HFpEF, heart failure with preserved ejection fraction. RV, right ventricle. HFD, high-fat diet. L-NAME, Nω-nitro-L-arginine methylester hydrochloride. MACS, magnetic-activated cell sorting. ns, not significant.

To decipher how macrophage accumulation contributes to RV remodeling in HFpEF, we next performed gene expression profiling in bulk RV tissue, MACS-sorted CD64^+^ macrophages and Ly6G^+^ neutrophils from RV tissue of RV-HFpEF mice, screening for pro-inflammatory and pro-fibrotic transcripts (Fig. 6A, B). Inflammatory mediators such as *tumor necrosis factor alpha* and *interleukin 1 beta* were particularly enriched in neutrophils from RV-HFpEF mice, while pro-fibrotic marker expression was associated with CD64^+^ macrophages, highlighting again the importance of innate immune cell populations in RV tissue remodeling in HFpEF.

Based on these data, we hypothesized that macrophages with a pro-fibrotic profile may be particularly relevant to RV remodeling and subsequent RVD development in HFpEF. To test this notion, we depleted the cardiac macrophage pool by pharmacologically targeting the colony-stimulating factor 1 receptor (CSF1R)^48^. CSF1R promotes macrophage differentiation, survival, and myeloid cell proliferation^49^, whereas CSF1R inhibition rapidly results in macrophage depletion of cell populations relying on CSF1R^48^. Macrophage depletion in RV-HFpEF mice was achieved by treatment with the CSF1R inhibitor PLX5622 for six weeks, starting six weeks after HFpEF induction (Fig. 6C). This experimental design was chosen based on the hypothesis that potential pro-fibrotic effects mediated by myeloid cells may only become effective at a later disease stage at the time of RVD development and when LV diastolic dysfunction is already established. Treatment with PLX5622 sufficiently depleted cardiac CD64^+^ macrophages below 2% of total cardiac leukocytes whereas blood leukocyte counts and LV function remained stable (Fig. 6D, E, Fig. S10A-G, Fig. S11A-C). Of note, we also found cardiac neutrophil counts reduced in RV tissue from macrophage-depleted RV-HFpEF mice, suggestive of macrophage-neutrophil interaction in HFpEF-associated RVD (Fig. 6F). While RV remodeling, as evidenced by RV hypertrophy and RV matrix deposition remained unchanged in response to macrophage depletion, RV hemodynamics, pro-inflammatory and pro-fibrotic gene expression patterns were rescued in RV-HFpEF mice upon macrophage depletion (Fig. 6G-J). To probe for potential indirect effects of macrophages on RV tissue via macrophage-neutrophil interactions, we employed a neutrophil depletion strategy using antibodies directed against neutrophil surface markers (Fig. S12A). Neutrophil depletion effectively reduced neutrophil abundance in RV tissue in RV-HFpEF mice (Fig. S12B), yet failed to rescue RV myeloid cell counts, RV hemodynamics, RV function and RV inflammatory profiles (Fig. S12C-I). Finally, we aimed to limit RV monocyte recruitment and thereby prevent RVD development in RV-HFpEF mice by employing pharmacological CCR2 inhibition (Fig. S13A). Unexpectedly, treatment with the potent CCR2 antagonist RS102895 did not reduce macrophage and CCR2^+^ macrophage counts in RV-HFpEF animals (Fig. S13B-G). This effect may be explained by a compensatory upregulation of other chemokine signaling pathways, namely the *Cxcr4/Cxcl12, Cx3cr1/Cx3cl1* and *Ccl5* axes, likely maintaining monocyte recruitment in conditions of CCR2 inhibition (Fig. S13H-J).

Our results indicate that macrophage depletion partially rescues RV phenotypes in RV-HFpEF mice, suggesting that myeloid cells are causally involved in RVD pathogenesis in HFpEF by directly mediating tissue inflammation and remodeling. Conversely, our results also reveal RV remodeling processes that are independent of myeloid cell accumulation in HFpEF associated RVD.

### RV dilation is associated with unique blood leukocyte signatures in HFpEF patients

**Fig. 7:**
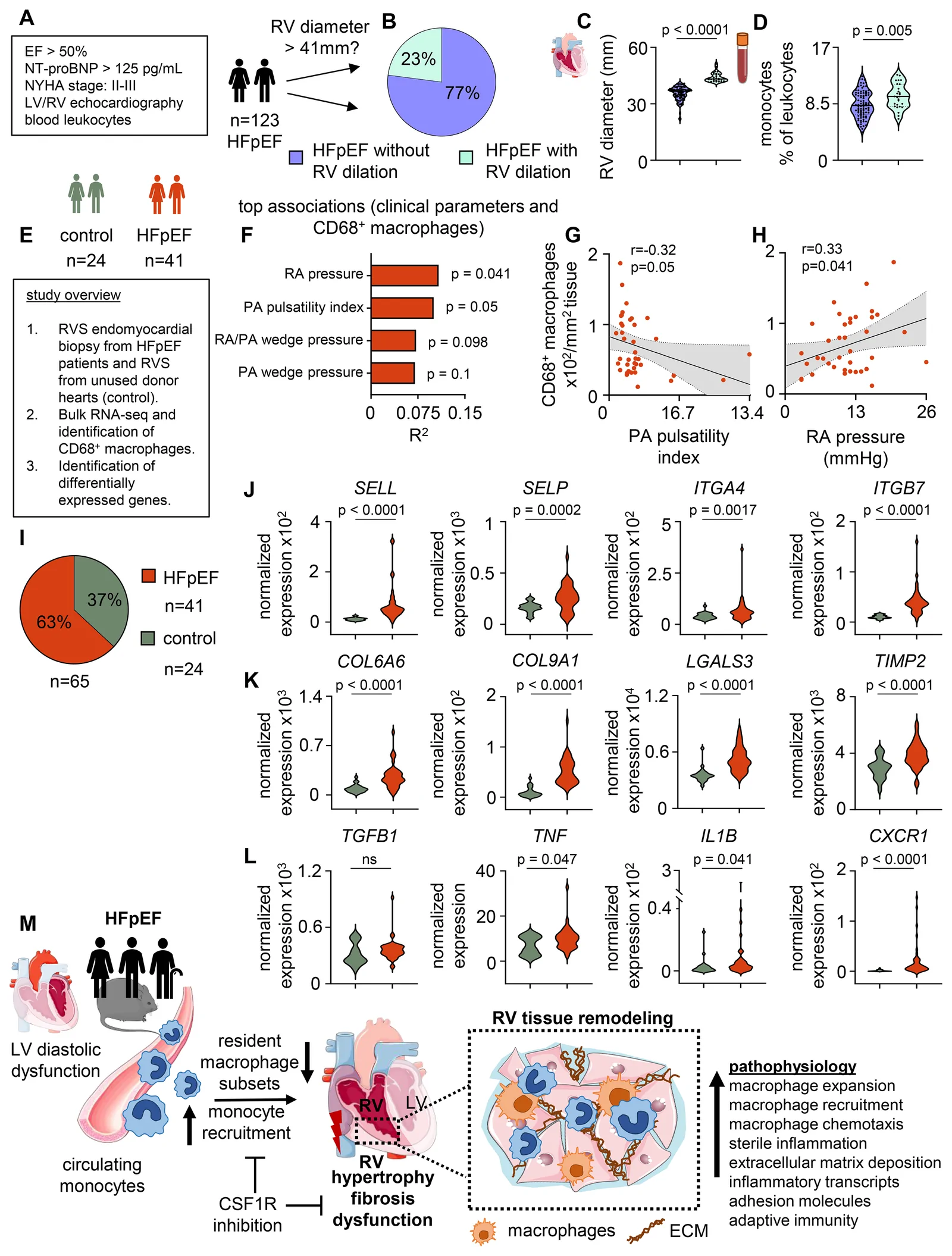
CD68^+^ cell counts from RV biopsies correlate with clinical signs of RV dysfunction. **A**, Experimental outline of patient cohort with HFpEF from Collaborative Research Center (CRC) 1470 (n=123). **B**, Percentage of HFpEF patients with and without RV dilation. **C**, Echocardiographic analysis of RV diameter HFpEF patients with and without RV dilation. **D**, Percentage monocytes of leukocytes in HFpEF with RV dilation and HFpEF without RV dilation (n=28–95). **E**, Study overview for the CD68^+^ macrophage quantification and identification of differentially expressed genes in RV septum (RVS) biopsies from HFpEF vs. controls. **F**, Top associations between available clinical parameters from HFpEF patients and CD68^+^ cell counts. **G**, Linear correlation analysis using Pearson’s r and 95% confidence interval between **G**, PA pulsatility index and CD68^+^ cells and **H**, RA pressure and CD68^+^ cells from HFpEF RVS biopsies. **I**, Data were pulled from a previously published data repository^24^. Percentage of HFpEF and control patients included for bulk RNA-seq of RVS biopsies. **J, K, L**, Differential expression of selected adhesion molecules (**J**), pro-fibrotic markers (**K**) and inflammatory markers (**L**) in RVS biopsies from control (n=24) vs. HFpEF (n=41) patients. P values were adjusted for multiple comparisons by using the Benjamini-Hochberg method. **M**, Graphical abstract. P values were calculated using a Mann–Whitney test (C), or a Wilcoxon rank sum test (D). HFpEF, heart failure with preserved ejection fraction. NYHA, New York Heart Association. RVS, RV septum. EF, ejection fraction. CSF1R, Colony stimulating factor 1 receptor. LV, left ventricle. RV, right ventricle.PA, pulmonary artery. RA, right atrium. ECM, extracellular matrix. ns, not significant.

To assess the relevance of our findings in the clinical setting, we retrospectively studied leukocyte counts in a consecutive HFpEF patient cohort recruited by our Collaborative Research Center 1470 (n=123, further referred to as ‘CRC1470 cohort’), which were included according to the ACC guidelines^50^ matching the following criteria: i) EF>50%, ii) N-terminal pro-B-type natriuretic peptide (NT-proBNP) >125 pg/mL, iii) New York Heart Association (NYHA) stage II or III, and iv) biventricular echocardiography (Fig. 7A). We dichotomized the CRC1470 patient cohort by RV dilation in HFpEF patients with RV dilation (>41mm) and HFpEF patients without RV dilation (<41mm). RV dilation is often associated with RVD and worse outcomes and according to a meta-analysis is the most sensitive measure predictive of mortality in HFpEF patients^44,51,52^. According to this stratification, 23% of HFpEF patients had RV dilation (Fig. 7B, C). The patient characteristics revealed that HFpEF patients with RV dilation were more frequently male, had higher NT-proBNP serum levels and had experienced atrial fibrillation more frequently compared to HFpEF patients with regular RV diameters (Table 1). Average sPAP, sPAP-TAPSE ratio, and right atrial dilation were all impaired in HFpEF patients with RV dilation, indicating signs of RVD. Interestingly, total leukocyte counts were significantly reduced in HFpEF patients with RV dilation when compared to HFpEF patients with normal RV function (Table 1). White blood cell count differentials revealed a myeloid cell bias towards increased percentages of monocytes in HFpEF patients with RV dilation (Fig. 7D), whereas neutrophils and lymphocyte counts remained comparable in both groups (Table 1).

### RV myeloid cell expansion associates with clinical signs of RVD

Finally, we asked whether RV macrophage-associated mechanisms identified in RV-HFpEF mice translate to the clinical scenario. The presence of CD68^+^ macrophages in endomyocardial RV biopsies from HFpEF patients has been previously described^24,53,54^, but it remains unclear to which extent RV septum (RVS) CD68^+^ macrophage expansion associates with RVD in HFpEF patients. To address this important question, we correlated histologically-assessed CD68^+^ cell counts from RVS biopsies with RV hemodynamic data from the same HFpEF patients (Fig. 7E). Interestingly, CD68^+^ cell counts correlate with right atrial pressure and pulmonary arterial pulsatility index (PAPi), but not with pulmonary artery systolic pressure profiles in HFpEF patients (Fig. 7F-H, Fig. S14A-C), indicating that RV myeloid cell expansion is associated with RVD and RV decompensation, but not necessarily with hemodynamic features related to pulmonary vascular load. In line, reanalysis of a published bulk RNA sequencing dataset from RVS endomyocardial biopsies from unused donor hearts (controls) and HFpEF patients^24^ (Fig. 7I) revealed i) increased expression levels of cell adhesion molecules associated with leukocyte-endothelial cell interaction such as selectins and integrins (*SELL, SELP, ITGA4, ITGB7*, Fig. 7J), ii) elevated expression of matricellular proteins and pro-fibrotic mediators such as collagens, galectins and tissue inhibitors of metalloproteinases (*COL6A6, COL9A1, LGALS3, TIMP2*, Fig. 7K), and iii) higher expression of pro-inflammatory cytokines and their receptors (*TNF, IL1B, CXCR1)*, while *TGFB1* was not significantly regulated (Fig. 7L), altogether replicating and thus, validating our murine data from RV-HFpEF mice in RV tissue from HFpEF patients.

## Discussion

Collectively, our data indicate that myeloid cells contribute to the pathogenesis of RVD in HFpEF (Fig. 7M). This is an important novel finding as ventricle-specific pathomechanisms have, as of yet, not been addressed in the context of HFpEF, despite the emerging recognition of monocyte-derived macrophages as a pathogenic factor of ventricular dysfunction in HFpEF^7,55^. Here, we identified macrophage chemotaxis, unique cell adhesion molecule profiles, monocyte expansion and recruitment, and extracellular matrix disorganization as mechanisms at play in RVD associated to HFpEF.

Hypoxemia is a common finding in HFpEF patients, in particular during exercise^29–31^. Almost half of the patients diagnosed with PH due to left heart disease also show features of secondary lung disease/hypoxemia, indicating significant overlap between group II and III PH^56^. Patients with HFrEF living at high altitude also have more major adverse cardiovascular events than lowlanders^57^, although similar data are lacking for HFpEF. While the combination of hypoxia and HFpEF is thus a relevant clinical scenario, it should be noted that the 10% chronic hypoxia used in our RV-HFpEF model exceeds typical clinical levels of hypoxia and is more acute than these chronic exposures. That notwithstanding, the present model provides proof-of-principle for synergistic effects of hypoxia and metabolic/hypertensive stress.

Our data from HFpEF patients identified lower leukocyte counts in HFpEF with RV dilation compared to HFpEF with normal RV diameters. Whilst leukocyte counts in HFpEF patients with RVD were lower, white blood cell counts were still in the upper physiological range. Reduced leukocyte counts have been reported before in patients with decompensated heart failure and in patients with idiopathic PAH^58,59^. This observation may be, at least in part, explained by our finding of increased expression of leukocyte adhesion molecules in RV tissue, which is associated with enhanced immune cell recruitment to the site of injury and could therefore explain lower levels of circulating white blood cells. Increased expression of adhesion molecules has been previously reported in clinical and experimental HFpEF^40,60,61^ as well as in PH and RV failure^62,63^, suggesting cell adhesion as a key feature in RVD-HFpEF. Another likely explanation for this finding may be a disturbed production or processing of mature leukocytes in the bone marrow of HFpEF individuals. This thought is supported by recent cumulative evidence, linking the risk of incident HFpEF to clonal hematopoiesis of indeterminate potential (CHIP)^64–66^. Data to test if the phenogroup of HFpEF patients with RVD are more frequently affected by CHIP or other hematopoietic disorders such as myelodysplastic syndrome when compared to HFpEF patients without RVD, are to our knowledge not yet available.

Besides the association of circulating leukocyte counts with RV dilation in HFpEF patients, studying leukocyte dynamics in the clinical scenario is restricted by the limited availability of RV tissue samples from HFpEF patients. CD68^+^ macrophages have been documented in endomyocardial RVS biopsies from HFpEF patients, with increased macrophage burden correlating with distinct clinical phenotypes and suggesting discrete HFpEF endotypes^24^. These data are further supported by our finding that macrophage accumulation correlated with RV function, but not with hemodynamic features related to pulmonary vascular load, supporting the overall concept of a clinical endotype of RVD-HFpEF with unique underlying mechanistic features such as myeloid cell expansion. This concept is reinforced by single-cell RNA sequencing, which identifies a macrophage subset in HFpEF-RVs characterized by a disease-specific transcriptional signature relative to controls^53^. Complementary proteomic analyses demonstrate significant enrichment of immune, cell-migration, adhesion, and chemotactic pathways in HFpEF RVS tissue, indicating a robust inflammatory component associated with myeloid-cell expansion^54^.

To overcome the limited availability of patient biopsies, we designed the novel murine ‘RV-HFpEF’ model, which develops clinical signs of both HFpEF and RVD. Interestingly, RV-HFpEF does not present with sexual dimorphism as described for the gold-standard two-hit HFpEF model^35^. Given that RV-HFpEF does not rely on genetic manipulation, it enables the combination with cell-specific loss-of-function approaches, e.g. by Cre/lox or Dre/rox systems to study cell-specific molecular mechanisms of RVD in HFpEF. Our model combines classic HFpEF triggers, namely metabolic stress and systemic hypertension, with hypoxia, thus mimicking reduced oxygen levels due to an increase in the alveolar-arterial O_2_ gradient in HFpEF patients^29,30,31,67^. Of note, the RV-HFpEF model is specifically tailored to match the clinical features of HFpEF patients developing RVD and hypoxic remodeling, rather than resembling the entire spectrum of HFpEF disease.

In our model, RV inflammation, but not RV matrix deposition, was reversed by CSF1R-mediated macrophage depletion, indicating that cells other than those relying on the myeloid receptor CSF1R contribute to matrix deposition in RV-HFpEF. Notwithstanding this, RV hemodynamics improved in response to macrophage depletion independent of collagen content. We speculate that mechanisms beyond collagen deposition, such as direct inflammatory effects impacting cardiomyocyte function, contribute to impaired RV hemodynamics in HFpEF. This notion is supported by the finding of reduced RV neutrophil counts in the absence of macrophages in RV-HFpEF mice. Although neutrophil depletion was not sufficient to rescue HFpEF-associated RVD in our mouse model, neutrophils from HFpEF patients have been shown to release more cytokines compared to controls and LV diastolic dysfunction has previously been linked to neutrophil degranulation and Neutrophil Extracellular Trap (NET) formation in HFpEF^68–70^. Moreover, depletion of resident macrophages via CSF1R inhibition has been shown to reduce neutrophil influx and neutrophil-mediated cytokine release, ultimately mitigating tissue injury^71,72^. Our results from macrophage depletion experiments link macrophage and neutrophil dynamics, indicating cellular crosstalk between these two immune cell populations in HFpEF-associated RVD. The fact that LV dysfunction was not rescued by macrophage depletion may, on the one hand, be attributable to our experimental design, as CSF1R inhibitor treatment was only initiated six weeks after HFpEF induction. On the other hand, this finding is coherent with the fact that HFpEF-LVs rely less on macrophage expansion than HFpEF-RVs. Future studies with preventive macrophage depletion approaches are necessary to study the role of macrophages in LV diastolic dysfunction.

Elevated macrophage counts in HFpEF LVs have been shown to be mediated by the interaction of C-C chemokine receptor type 2 (CCR2) with its ligand monocyte chemoattractant protein-1 (CCL2), which effectively regulate monocyte chemotaxis. Consistently, genetic inhibition of the CCR2/CCL2 axis prevented cardiac macrophage expansion and improved diastolic function in a murine model of angiotensin-II induced HFpEF^55,73^. In our study we found macrophage expansion via recruitment and proliferation of CCR2^+^ myeloid cells in HFpEF RVs at the expense of TIMD4^+^ and LYVE1^+^ resident macrophage subsets. In contrast to recruited macrophage subsets, which promote extracellular matrix deposition after tissue injury^7^, TIMD4^+^ and LYVE1^+^ resident macrophage subsets protect from cardiac tissue remodeling and ventricular dysfunction^74–76^. Our findings are in line with recently published literature on the role of macrophages in LV diastolic dysfunction which demonstrate that resident macrophage subsets are cardioprotective in HFpEF^77,78^, whereas CCR2^+^ monocyte-derived macrophages drive cardiac hypertrophy in HFpEF and are associated with increased extracellular matrix deposition and fibroblast activation, thus promoting myocardial stiffness and impairing myocardial relaxation^7,79^. Of note, modulating CCR2^+^ macrophage subsets by vagal stimulation prevents tissue inflammation and fibrosis in HFpEF LVs^77,80^. In our study, CCR2 inhibition did not reduce monocyte recruitment in RV tissue from RV-HFpEF mice, but increased expression profiles of other chemokine signaling pathways, consistent with other animal models involving hypoxic exposure^81^. These data highlight the complexity of multi-hit animal models mimicking the clinical HFpEF syndrome and demonstrate the limitations of single-pathway interventions in multifactorial disease processes such as immune cell recruitment into injured tissues. While macrophage-associated RV remodeling and RVD have been implicated in several forms of PAH^62,63,82^, our study is the first to demonstrate ventricle-specific differences in macrophage expansion and recruitment in RV-HFpEF, highlighting the need to study disease mechanisms in HFpEF individually for cardiac chambers.

In aged animals, we found increased macrophage accumulation independent from HFpEF, which was particularly attributable to a greater proportion of the CCR2^+^MHCII^hi^ macrophage subset. CCR2^+^MHCII^hi^ cardiac macrophages are a monocyte-derived, CCR2-dependent population that expands in the failing heart and exhibits a pro-inflammatory phenotype^76,83,84^, thereby establishing RV myeloid cell expansion as a mechanistic feature of biological aging. Moreover, in aged mice undergoing RV-HFpEF, total macrophages and MHCII^hi^ macrophage subsets were enriched independent of CCR2 expression and associated with accelerated RV remodeling and RVD. In line, increased MHCII expression on myeloid cells has been described before in humans and mice of increased biological age and has been linked to distinct immunological functions^85–87^. Together, these data highlight RV inflammation as an age-associated contributor to RVD in HFpEF and may serve as an explanation why RVD-HFpEF is frequently observed in older patients.

## Conclusion

Our translational work shows that dysregulated leukocyte counts and myeloid cell expansion are associated with, and directly contribute to, the pathogenesis of HFpEF-associated RVD. We demonstrate that LV and RV exhibit distinct inflammatory responses, when exposed to the same systemic disease triggers in HFpEF, with HFpEF-RVs being particularly vulnerable to myeloid cell recruitment and sterile inflammation compared to HFpEF-LVs. Immunomodulation of myeloid cells may therefore provide a potential therapeutic approach to prevent RV remodeling and dysfunction in HFpEF.

## Limitation

Our study is limited by the availability of fresh human RV free wall tissue for the validation of leukocyte accumulation in HFpEF patients. Our study lacks experiments selectively blocking monocyte recruitment in RV-HFpEF mice, as the employed approach of pharmacological CCR2 inhibition failed to reduce monocyte recruitment by compensatory upregulation of other chemotactic pathways.

## Supplementary Material

ARRIVE_guideline

Excel_S1

Excel_S2

Supplementary Material

## Figures and Tables

**Table 1:** Baseline characteristics of CRC1470 patient cohort.

Characteristics	HFpEF Without RV dilation	HFpEF with RV dilation	P-value
**HFpEF**			
N (%)	95 (77%)	28 (23%)	
Age (years)	75 (50, 87)	76 (61, 89)	0.886
Female gender (%)	55 (58%)	9 (32%)	0.017
Male gender (%)	40 (42%)	19 (68%)	
NYHA class II (%)	66 (69%)	18 (64%)	0.6
NYHA class III (%)	29 (31%)	10 (36%)	
NT-pro-BNP (pg/mL)	395 (128, 2412)	499 (154, 2047)	0.062
LV EF (%)	57.0 (45.0, 74.0)	54.0 (50.0, 60.0)	0.11
**RV function**			
RV diameter	36.64 (22.00, 40.90)	43.25 (41.41, 51.23)	<0.0001
sPAP (mmHg)	31 (16, 72)	38 (20, 57)	0.002
TAPSE/sPAP Ratio	0.74 (0.37, 1.69)	0.59 (0.29, 1.20)	0.012
RA dilation	47 (53%)	21 (88%)	0.002
**Comorbidities**			
Hypertension	75 (79%)	23 (82%)	0.7
Atrial Fibrillation	54 (57%)	24 (86%)	0.005
Diabetes Mellitus Type 2	20 (21%)	5 (18%)	0.7
Congestion/Edema	23 (25%)	9 (32%)	0.4
**Inflammation**			
Leukocyte counts (×10^3^/µL)	7.00 (3.82, 13.80)	6.56 (3.19, 9.62)	0.029
Monocyte %	8.20 (4.20, 12.60)	9.60 (6.20, 12.90)	0.005
Neutrophil %	65 (44, 83)	65 (45, 78)	0.5
Lymphocyte %	24 (5, 41)	22 (13, 41)	0.2

HFpEF, Heart failure with preserved ejection fraction, NYHA, New York Heart association, NT-pro-BNP, N-Terminal Pro-B-Type Natriuretic Peptide, LVEF, Left ventricular ejection fraction, RV, right ventricle S’, TAPSE, tricuspid annular plane systolic excursion, sPAP, systolic pulmonary artery pressure, RA, right atrium CRP. P-values calculated using a Wilcoxon rank sum test, Fisher’s exact test or Pearson’s Chi-squared test. Data are presented as median with range from min to max or n (%).

## Clinical Perspective

### What is new

- We explore myeloid cell dynamics in a novel three-hit experimental HFpEF mouse model with RV hypertrophy, RV end-systolic pressure and RVD.
- In this model, RVD was associated with macrophage expansion, monocyte recruitment and extracellular matrix deposition, whilst macrophage depletion partly reversed these changes and rescued RV hemodynamics.
- HFpEF patients with RV dilation or RVD exhibit unique leukocyte dynamics and inflammatory profiles when compared to HFpEF patients with normal RV function or diameters.

### Clinical implications

- There exists a major clinical discrepancy between high incidence of RVD associated to HFpEF and a lack of targeted treatment strategies.
- Our novel three-hit mouse model recapitulates key features of a specific clinical endotype of HFpEF patients developing RVD, therefore representing an important step towards systematic testing and development of targeted treatment options for a subset of patients with particularly adverse outcome.
- Myeloid cell counts in RV biopsies are associated with worse RV hemodynamics in HFpEF patients and may therefore be suitable targets for therapeutic interventions against RVD-HFpEF.

## Non-standard Abbreviations and Acronyms

CCL2: C-C motif chemokine ligand 2
CCR2: C-C motif chemokine receptor 2
CHIP: clonal hematopoiesis of indeterminate potential
COPD: chronic obstructive pulmonary disease
CRC1470: Collaborative Research Center 1470
CSF1R: colony-stimulating factor 1 receptor
CX3CR1: C-X3-C motif chemokine receptor 1
EF: ejection fraction
GLS: global longitudinal strain
GSEA: gene set enrichment analysis
HFD: high-fat diet
HFpEF: heart failure with preserve ejection fraction
HFrEF: heart failure with reduced ejection fraction
LAVI: left atrial volume index
L-NAME: N[ω]-nitro-l-arginine methyl ester
LV: left ventricle
LVMI: left ventricular mass index
NET: neutrophil extracellular trap
NT-proBNP: N-terminal pro-B-type natriuretic peptide
NYHA: new york heart association
PA: pulmonary artery
PAH: pulmonary arterial hypertension
PAP: pulmonary artery pressure
PAPi: pulmonary artery pulsatility index
PASP: pulmonary artery systolic pressure
PH: pulmonary hypertension
RA: right atrium
RV: right ventricle
RVD: right ventricular dysfunction
RVS: right ventricular septum
RWT: relative wall thickness
TAPSE: tricuspid annular plane systolic excursion
