# Supplementary material for: Myeloid cell expansion propels right ventricular dysfunction in HFpEF via sterile inflammation
Source: Circulation. Author manuscript; Available in PMC 2026 Sep 29. (PMC7619445; doi:10.1161/CIRCULATIONAHA.125.078476)
Supplement: Supplementary file 2 [file EMS216731-supplement-Supplemental_Material.pdf]

SUPPLEMENTAL MATERIAL

Myeloid cell expansion propels right ventricular dysfunction in HFpEF via sterile inflammation.

Lara Jaeschke, MS[1,2,3,4], Ceren Koçana, MS[1,2,3,4], Alexandra Maria Chitroceanu, MD[2,4,5], Annika Winkler, MS[1,2,3], Hannah Kleitke, MS[1,2,3,4], Pauline Fahjen, MS[6], Justus Kamp, student [1,2,3,4,7], David Faidel, student, [1,2,3,4], Erik Asmus, student, [1,2,3,4], Martin Meiser, student[1,2,3], Efstathios G Stamatiades, PhD[2,8], Karolin W. Hublitz, MS[2,8], Veronika Zach, MD[4,9], Lucie Kretzler MD[9], Daniela Zurkan, MD[2,4,5], Paul-Lennard Perret, MD[2,3,4,10], Leonardo A. von der Ohe, student[1,2,3,4], Kai Beckschulte, student[1,2,4], Szandor Simmons, PhD[2,3,4], Jonathan L. Gillan, PhD[1,2,3,4], Kristina Franz, PhD[2,4,5,11,12], Lina Alasfar, PhD[10,13,14], Virginia S. Hahn, MD[15], Kavita Sharma, MD[15], Edwardo Reynolds, PhD[2,5], Gabriele G. Schiattarella, MD, PhD[4,16,17,18,19,20], Sophie van Linthout, PhD[4,5,10], Burkert Pieske, PhD[13], Niklas Beyhoff, MD, PhD[1,2,4], Christiane Ott, PhD[4,5], Niklas Hegemann, PhD[1,2,3,4], Philipp Mertins, PhD[2,4,6], Frank Edelmann, MD[2,4,5], Wolfgang M. Kuebler, MD[3,4,21]*, Jana Grune, PhD[1,2,3,4,21]*#

Supplemental Methods

Humans

Study design

To detect an effect in terms of a hazard ratio of 0.6 with power of 80% at a two-sided significance level of 5%, a total of 120 patients must show the event of interest (RV dilation). To assess RV dilation, n = 123 patients were included. n=3 were missing a monocyte measurement. Potential confounders are sex, age, body mass index, and relevant comorbidities. We did not control for confounders. Data were obtained from standardized clinical phenotyping and biosampling while echocardiographic data were acquired as part of the baseline assessment and laboratory data were obtained from venous blood samples collected during the baseline assessment. Quantitative variables were analyzed primarily on their continuous scale to preserve information. Potential selection bias was addressed by applying pre-specified inclusion and exclusion criteria and documenting screening, eligibility, consent, and participation status. Information bias was reduced through standardized operating procedures, trained study personnel, structured electronic data capture, predefined variable definitions, and plausibility checks during data curation.

Assessment of LV function

Conventional transthoracic echocardiography (TTE) was performed using a commercially available ultrasound system (Vivid E95, GE Vingmed Ultrasound AS, Horten, Norway) equipped with a 3.5 MHz transducer. Cardiac chamber size and function were quantified following the current guideline recommendations[88]. LVEF was calculated using the modified Simpson's rule. LVMI was derived from the parasternal long-axis view using the linear method (cube formula) and indexed to body surface area (BSA). RWT was calculated as 2 x posterior wall thickness relative to left ventricular internal diameter at end-diastole. Left atrial volume index (LAVI) was determined using the biplane disk summation technique and indexed to BSA. Tissue Doppler imaging was used to record the velocities of the septal and lateral mitral annulus, and their mean (E') was calculated. Early diastolic mitral inflow peak velocity (E wave) was measured, and the E/E' ratio was calculated (data not shown).

Assessment of RV function

RV measurements were performed using a dedicated RV-focused apical four-chamber view in accordance with current guidelines[88]. The RV basal linear diameter was measured as the maximal transversal dimension in the basal one-third of the RV inflow at end-diastole. RV systolic function was evaluated using multiple parameters, including tricuspid annular plane systolic excursion (TAPSE) and tissue Doppler velocity tricuspid annular systolic velocity (S'). TAPSE was measured using M-mode echocardiography with the cursor optimally aligned along the direction of the tricuspid lateral annulus in the apical four-chamber view. Tissue Doppler velocity tricuspid annular systolic velocity (S') was obtained from the apical approach, ensuring alignment of the Doppler beam parallel to the longitudinal excursion of the RV free wall. All echocardiographic parameter cutoff values were set according to current guidelines. A RV diameter of > 41 mm basal diameter was considered abnormal and used as indicator for RVD[88]. HFpEF patients were accordingly stratified into two groups: those with RV diameter > 41mm and those with normal RV diameter.

Mice

Health monitoring

All mice underwent body weight monitoring twice a week and health monitoring twice daily. Behavior and phenotypic appearance were checked for *a priori*-defined humane endpoints. None of the humane endpoints were reached in experiments reported within this study. A regularly occurring symptom was

body weight loss up to 25% in response to hypoxic exposure, but was tolerated due to *a priori*-defined humane endpoint (weight deficit of ≥30% compared to its own weight). No animals were excluded from experiments or data analysis.

Macrophage depletion

Macrophages were depleted by feeding mice with the colony stimulating factor 1 receptor (CSF1R) inhibitor PLX5622 (PLX, 1200 ppm., Hycultec GmbH) compressed in high-fat diet for six weeks to deplete macrophages.

CCR2 inhibition

CCR2 was inhibited by administration of the selective CCR2 antagonist RS-102895 (MedChemExpress, RV-HFpEF+CCR2i) or vehicle (DMSO) to RV-HFpEF (RV-HFpEF+vehicle), via drinking water (10 mg/kg/day) for the final 2 weeks of the experiment.

Neutrophil depletion

Neutrophils were depleted by injections of anti-mouse Ly6G (clone 1A8, 50µg/d/mice, BP0075-1, BioXcell, RV-HFpEF+neutrophil depletion) and anti-rat Kappa Immunoglobulin Light Chain antibody (clone MAR 18.5, 50µg/d/mice, BE0122, BioXcell) for the last 2 weeks of the experiment[89]. Control mice were injected with rat IgG2a (clone 2A3, 50µg/d/mice, BP0089, BioXcell) and mouse IgG2a isotype control (clone C1.18.4, 50µg/d/mice, BE0085, BioXcell).

Fate mapping experiments

For activation of the Cre/loxP system, 8-12-week-old *Cx3cr1*$^{CreER/+}$*R26*$^{tdTomato/+}$ mice received tamoxifen-containing diet for 7 days (265 mg kg−1 diet, Ssniff), starting four weeks prior to HFpEF induction. tdTomato expression in circulating $CD115^+$ monocytes was confirmed by flow cytometric analyses of blood samples taken after tamoxifen (week -3) exposure and after tamoxifen wash out (week 0).

Blood gas analysis

For hemoglobin and hematocrit measurements, 70 µl whole blood were analyzed with an i-STAT CG8+ Cartridge using the i-STAT 1 system (both Abbott).

Gravimetry

Peripheral mouse blood was collected by retroorbital bleeding using heparinized capillary tubes (BRAND GMBH+CO KG, Wertheim, Germany). Hearts were perfused through the left ventricle with 10 mL ice-cold 1X PBS, excised, separated from atria, divided into LV (septum included) and RV and weighed. For gravimetric analysis and calculation (Fulton’s index, RV/TL, RV/HW), tibias were removed and measured.

Flow cytometry

Whole blood: Red blood cells were lysed with 1x red blood cell lysis buffer (BioLegend). For fate mapping experiments. red blood cells were stained at 4 °C for 30 min in MACS separation buffer (PBS, EDTA, BSA and 0,09% Azide, Miltenyi Biotec) with CD45-BV711 (30-F11, 1:1000, 103147, BioLegend), CD11b-Pe-Cy7 (clone M1/70, 1:1000, 101216, BioLegend), CD3-Biotin (clone 17A2, 1:300, 100244, BioLegend), CD19-Biotin (clone 6D5, 1:1000, 115503, BioLegend), B220-Biotin (clone RA3–6B2, 1:100, 103204, BioLegend), Nk1.1-Biotin (clone PK136, 1:100, 108704, BioLegend), Ly6G-APC (clone 1A8, 1:600, 127614, BioLegend), CD115-BV605 (clone AFS98, 1:600, 135517, BioLegend) and Streptavidin-APC-Cy7 (1:250, 405208, BioLegend) antibodies. Viable cells were detected as negative for DAPI staining (BD Bioscience, 564907). Leukocytes were defined as $CD45^+$ cells. Monocytes were defined as $CD45^+$ $(CD3/CD19/B220/Nk1.1)^-$ $CD11b^+$ $Ly6G^-$ $CD115^+$ cells and were further analysed for tdTomato expression (Figure S9D).

For macrophage depleting experiments red blood cells were stained at 4 °C for 30 min in MACS separation buffer (PBS, EDTA, BSA and 0,09% Azide, Miltenyi Biotec) with CD45-BV711 (30-F11, 1:600, 103147, BioLegend), CD11b-APC-Cy7 (clone M1/70, 1:600, 101226, BioLegend), CD3-PE (clone 17A2, 1:200, 100206, BioLegend), CD19-PE (clone 6D5, 1:300, 115507, BioLegend), B220-PE (clone RA3–6B2, 1:300, 103207, BioLegend), Nk1.1-PE (clone PK136, 1:300, 108707, BioLegend), Ly6G-APC (clone 1A8, 1:600, 127614, BioLegend), CD115-BV605 (clone AFS98, 1:600, 135517, BioLegend) and Ly6C-PerCP-Cy5.5 (clone HK1.4, 1:600, 128012, BioLegend) antibodies. Viable cells were detected as negative for DAPI staining (BD Bioscience, 564907). Leukocytes were defined as $CD45^+$ cells. Neutrophils were defined as $CD45^+$ $(CD3/CD19/B220/Nk1.1)^-$ $CD11b^+$ $CD115^-$ $Ly6G^+$ cells. Monocytes were defined as $CD45^+$ $(CD3/CD19/B220/Nk1.1)^-$ $CD11b^+$ $Ly6G^-$ $CD115^+$ cells (Figure S9D).

Heart tissue: Ventricles were separated and minced into small pieces. Heart tissue was subjected to enzymatic digestion using collagenase I (450 U/ml), collagenase XI (60 U/ml), DNAse I and hyaluronidase (60 U/ml) (Sigma-Aldrich) buffered in HEPES buffer (Corning) and 1x PBS for 1 h at 37°C and 700 rpm. Cells were filtered through a 40-µm nylon mesh, washed, and centrifuged (5 min, 350xg, 4°C) to obtain single-cell suspensions. Cardiac cell suspensions were stained at 4°C for 30 min with CD45-PerCP-Cy5.5 (clone 30-F11, 1:600, 103132, BioLegend), CD11b-BV510 (clone M1/70, 1:600, 101245, BioLegend), Ly6C-AlexaFluor488 (clone HK1.4, 1:600, 128006, BioLegend), Ly6G-PE-Cy7 (clone 1A8, 1:600, 127617, BioLegend), CD64-APC (clone X54–5/7.1, 1:600, 139305, BioLegend), MHCII-APC-Cy7 (clone M5/114.15.2, 1:600, 107628, Biolegend) and CCR2-BV605 (clone SA203G11, 1:300, 150615, BioLegend) antibodies. Viable cells were detected as negative for DAPI staining (BD Bioscience, 564907). Leukocytes were defined as $CD45^+$ cells. Neutrophils were defined as $CD45^+$ $CD11b^+$ $Ly6G^+$ cells. Monocytes were defined as $CD45^+$ $Ly6G^-$ $CD11b^+$ $CD64^-$ $Ly6C^{hi}$ cells. Macrophages were defined as $CD45^+$ $Ly6G^-$ $CD11b^+$ $Ly6C^{lo}$ $CD64^+$ cells (Fig. 4J, Fig. 5B, G, Fig. 6D, Fig. S5A, Fig. S13B).

For Ki67 staining, cardiac single cell suspensions were labeled with CD45-BV711 (clone 30-F11, 1:300, 103147, BioLegend), CCR2-BV785 (clone SA203G11, 1:200, 150621, BioLegend), and Ly6C-AlexaFluor488 (clone HK1.4, 1:600, 128021, Biolegend) antibodies. Viable cells were detected with ZombieRed (1:1000, 423109 Biolegend). After fixation (30 min at 4°C) with fixation buffer cells were permeabilized with permeabilization buffer (00-5523-00, ThermoFisher Scientific). This was followed by one centrifugation step (5 min, 1300 rpm, 4°C) and a second staining with CD11b-APC-Cy7 (clone M1/70, 1:600, 101226, BioLegend), CD64-APC (clone X54-5/7.1, 1:300, 139305, BioLegend), Ly6G-PE/Cy7 (clone 1A8, 1:600, 127618, BioLegend) and Ki67-PE (clone 16A8, 1:300, 652403, BioLegend) antibodies in permeabilization buffer for 30 min at room temperature. As isotype control PE rat IgG2 (clone RTK2758, 1:300, 400507, BioLegend) antibody was used. DAPI was used to determine DNA content. Resting (G0) macrophages were identified as $CD45^+$ $CD11b^+$ $Ly6C^{lo}$ $CD64^+$ $Ki67^-$ $DAPI^-$ cells. Proliferating (G1) macrophages were identified as $CD45^+$ $CD11b^+$ $Ly6C^{lo}$ $CD64^+$ $KI67^+$ $DAPI^-$ cells. Dividing (S/G2/M) macrophages were identified as $CD45^+$ $CD11b^+$ $Ly6C^{lo}$ $CD64^+$ $Ki67^+$ $DAPI^+$ cells (Fig S9A).

For fate mapping experiments, cardiac cell suspensions were stained at 4°C for 30 min with CD45-BV711 (30-F11, 1:1000, 103147, BioLegend), CD11b-PeCy7 (clone M1/70, 1:1000, 101216, BioLegend), Ly6C-AlexaFluor488 (clone HK1.4, 1:600, 128006, BioLegend), Ly6G-PerCP-Cy5.5 (clone 1A8, 1:300, 127616, BioLegend), CD64-APC (clone X54–5/7.1, 1:300, 139305, BioLegend), MHCII-APC-Cy7 (clone M5/114.15.2, 1:600, 107628, Biolegend) and CCR2-BV785 (clone SA203G11, 1:600, 150621, BioLegend) antibodies. Viable cells were detected as negative for DAPI staining (BD Bioscience, 564907). Leukocytes were defined as $CD45^+$ cells. Monocytes were defined as $CD45^+$ $Ly6G^-$ $CD11b^+$ $CD64^-$ $Ly6C^{hi}$ cells. Macrophages were defined as $CD45^+$ $Ly6G^-$ $CD11b^+$ $Ly6C^{lo}$ $CD64^+$ cells and were further analyzed for tdTomato expression to define resident and recruited macrophages (Figure S9D).

For time course analyses of innate immune cells or analysis of adaptive immune cells, cardiac cell suspensions were stained at 4°C for 30 min with CD45-BV711 (30-F11, 1:400, 103147, BioLegend), Ly6C-AlexaFluor488 (clone HK1.4, 1:800, 128006, BioLegend), CD64-APC (1:400), TIMD4-BV786 (clone RMT4-45, 1:400, 744631, BD Bioscience), CCR2-RB780 (clone 475301, 1:400, 755695, BD Bioscience), LYVE1-PE (clone ALY7, 1:400, 12-0443-82, Invitrogen), MHCII-APC-Cy7 (clone M5/114.15.2, 1:1000, 107628, Biolegend), Ly6G-PerCP-Cy5.5 (clone 1A8, 1:800, 127616, Biolegend), NK1.1-PE-Cy5 (clone PK136, 1:400, 108716, Biolegend), CD19-Pe-Cy7 (clone 6D5, 1:1000, 115519, Biolegend) and CD3-R718 (clone 500A2, 1:100, 567303, BD Bioscience) antibodies. Viable cells were detected as negative for DAPI staining (BD Bioscience, 564907). Leukocytes were defined as $CD45^+$ cells. Monocytes were defined as $CD45^+$ $CD64^-$ $Ly6C^{hi}$ cells. Macrophages were defined as $CD45^+$ $Ly6C^{lo}$ $CD64^+$ cells. Resident $TIMD4^+$ macrophages were defined as $CD45^+$ $Ly6C^{lo}$ $CD64^+$ $CCR2^-$ $TIMD4^+$ cells. Resident $LYVE1^+$ macrophages were defined as $CD45^+$ $Ly6C^{lo}$ $CD64^+$ $CCR2^-$ $LYVE1^+$. Neutrophils were defined as $CD45^+$ $NK1.1^-$ $Ly6G^+$ cells. T-Lymphocytes were defined as $CD45^+$ $CD19^-$ $CD3^+$ cells. B-Lymphocytes were defined as $CD45^+$ $CD3^-$ $CD19^+$ cells. Natural killer cells were defined as $CD45^+$ $Ly6G^-$ $NK1.1^+$ cells (Fig. S4B, H).

We recorded three million events per sample using a Symphony A5 flow cytometer with FACSDiva 8.0.1 software and analyzed with FlowJo 10.8.1 software (BD Biosciences).

MACS cell separation

For $CD64^+$ isolation ventricles were separated and minced into small pieces. Heart tissue was subjected to enzymatic digestion using collagenase I (450 U/ml), collagenase XI (60 U/ml), DNAse I and hyaluronidase (60 U/ml) (Sigma-Aldrich) buffered in HEPES buffer (Corning) and 1x PBS for 1 h at 37°C and 700 rpm. Cells were filtered through a 40-µm nylon mesh and carefully layered over 5 mL of PBMC 24+ Spin Medium (1:1, pluriSelect). After centrifugation (20min, 350xg, RT), the mononuclear cell layer was again filtered followed by another centrifugation step (10 min, 350xg, 4°C). Cell suspensions were stained at 4°C for 15 min with CD64 APC (clone X54–5/7.1, 1:300, 139305, BioLegend), washed and centrifuged (10 min, 350xg, 4°C). For labeling and separation, cells were incubated for 15 min at 4°C with Anti-APC MicroBeads (130-090-855, 1:15, Miltenyi). After washing and centrifugation (10 min, 350xg, 4°C) $CD64^+$ cells were separated (positive selection) by transferring to MACS LS columns (Miltenyi) attached to a magnetic field. Columns were washed and after centrifugation (10 min, 350xg, 4°C) cells were stored at -80°C for further use.

For $Ly6G^+$ isolation ventricles were separated and minced into small pieces. Heart tissue was subjected to enzymatic digestion using collagenase I (450 U/ml), collagenase XI (60 U/ml), DNAse I and hyaluronidase (60 U/ml) (Sigma-Aldrich) buffered in HEPES buffer (Corning) and 1x PBS for 1 h at 37°C and 700 rpm. Cells were filtered through a 40-µm nylon mesh and carefully layered over 5mL of PolymorphPrep (1:1, PROGEN). After centrifugation (20min, 500xg, RT), the neutrophil layer was filtered followed by centrifugation (10 min, 350xg, 4°C). Cell suspensions were stained at 4°C for 15 min with Ly6G APC (clone 1A8, 1:300, 127614, BioLegend), washed and centrifuged (10 min, 350xg,

4°C). For labeling and separation, cells were incubated for 15 min at 4°C with Anti-APC MicroBeads (130-090-855, 1:15, Miltenyi). After washing and centrifugation (10 min, 350xg, 4°C) Ly6G$^+$ cells were separated (positive selection) by transferring to MACS LS columns (Miltenyi) attached to a magnetic field. Columns were washed and after centrifugation (10 min, 350xg, 4°C) cells were stored at -80°C for further use.

RNA isolation, cDNA synthesis and RT-qPCR

RNA from MACS separated cells was isolated using the RNeasy Micro Kit (74004, Qiagen) following the manufacturer's instructions. RNA from snap-frozen RV tissue was isolated by Trizol Reagent (Thermo Fisher). Tissue in trizol was disrupted using Zirkonia beads (2mm diameter; N039.1, Roth) and a FastPrep F120. RNA was further isolated by subsequent chloroform (Roth) extraction and precipitated using isopropanol (Roth). cDNA synthesis was performed using the Transcriptor First Strand cDNA Synthesis Kit (4896866001, Roche) following manufacturer's instructions. Quantitative real time PCR was performed with the FastStart Essential DNA Green Master (6402712001, Roche) using a QuantStudio5 (appliesBiosystems; ThermoFisherScientific). Expression values were normalized to the reference gene succinate dehydrogenase complex flavoprotein subunit A (*Sdha)* using the $2^{-\Delta\Delta Ct}$ method and presented as fold change relative to experimental controls. For the comparison of gene expression between RV bulk tissue and MACS sorted CD64$^+$ macrophages and Ly6G$^+$ sorted neutrophils as well as between male vs female expression values were calculated relative to *Sdha* expression using the $2^{-\Delta Ct}$ method. To quantify gene expression the following primers were used (annotated for each gene, forward and revers primer in 5'-3' orientation):

- *Spp1* (GCTTGGCTTATGGACTGAGGTC; CCTTAGACTCACCGCTCTTCATG),
- *Lgals3* (CTGCTGGCCCTTATGGTGTC; GTTTGCGTTGGGTTTCACTGT),
- *Timp1* (GCAACTCGGACCTGGTCATAA; CGGCCCGTGATGAGAAACT),
- *Il1b (*GAAATGCCACCTTTTGACAGTG; TGGATGCTCTCATCAGGACAG),
- *Tnf* (CATCTTCTCAAAATTCGAGTGACAA; TGGGAGTAGACAAGGTACAACCC),
- *Tgfb (*CTTCAATACGTCAGACATTCGGG; GTAACGCCAGGAATTGTTGCTA),
- *Myh7* (GGCCCTTTGACCTCAAGAAAG; CCGGATTCTCCGGTGATGAG),
- *Nppb* (CTGGGAAGTCCTAGCCAGTC; ACAACTTCAGTGCGTTACAGC),
- *Nppa* (GGCAGAGACAGCAAACATCA; ACAGTGGCAATGTGACCAAG),
- *Ccn2* (GAGTGTGCACTGCCAAAGATG; CAGGCTTGGCGATTTTAGGTG),
- *Col1a1* (TGTTCAGCTTTGTGGACCTC; GGTCCCTCGACTCCTACATCT),
- *Col3a1* (ACCTGGTTTCTTCTCACCCTTC; AAGGCCAGGGTCACCATTTC),
- *Cxcl1* (GGCGCCTATCGCCAATGAG; TACTTGGGGACACCTTTTAGC),
- *Cxcl2* (ATGGCCCCTCCCACCTG; CAGTTAGCCTTGCCTTTGTTCA),
- *Ccr5* (GGTTCCTGAAAGGCGGCTGTAAATA, CTGTTGGCAGTCAGGCACATC),
- *Ccl5* (AGAGGACTCTGAGACAGCACA, GCGGTTCCTTCGAGTGACAA),
- *Ccr1* (ACCTGTAGCCCTCATTTCCC, CAAATATCAGACGCACGGCT),
- *Cx3cr1* (GAAGTTCCCTTCCCATCTGCT, ATATGCCCCCAAAGAAGCCAAT),
- *Cxcl12* (GTCAGCCTGAGCTACCGATG, GCCCCTTGTTTAAAGCTTTCTC),
- *Cxcr4* (TTTGGTGCTCCGGTAACCAC, TGAAGGCCAGGATGAGAACG),
- *Cx3cl1* (ACGAAATGCGAAATCATGTGC, CTGTGTCGTCTCCAGGACAA),
- *Cx3cr1* (GAAGTTCCCTTCCCATCTGCT, ATATGCCCCCAAAGAAGCCAAT),
- *Sdha* (GGAACACTCCAAAAACAGACCT, CCACCACTGGGTATTGAGTAGAA).

BNP ELISA

Heparinised whole blood was centrifuged (15 min, 1000xg, 4°C) and plasma was obtained and analyzed using the mouse NT-proBNP ELISA Kit (ELK5445, ELK Biotechnology). Measurements were

conducted using the GloMax Plate Reader (Promega) and analysis was performed using a 5-parametric logistic regression.

Detection of triglycerides in liver homogenates

70-100 mg of fresh liver tissue was extracted from the animal, washed in cold PBS, dried on a paper towel, weighed, snap frozen in liquid nitrogen and stored at -80°C until analysis. For determination of triglyceride content, liver pieces were defrosted and absolute Ethanol was added in a ratio: 9ml/1g tissue. Samples were homogenzied with beads (Zirkonia Beads 2.0mm diameter (N039.1, Roth)) in 2ml screw-tubes (72.694.006, Sarstedt) by using the FastPrep FP120, program 4 for 3 times 20sec. Following centrifugation (15min, 10000xg 4°C), the supernatant was submitted to the Triglyceride (TG) Colorimetric Assay Kit (Single Reagent, GPO-PAP Method, EEA028, Invitrogen) following the manuals instructions.

Proteomics

Sample preparation for proteomic tissue analysis: For proteomic analysis, snap-frozen LV and RV samples were homogenized using a CryoPrep system (Covaris), resuspended in lysis buffer (1% Sodium deoxycholate (SDC), 150 mM NaCl, 100 mM Tris pH 8, 1 mM EDTA, 1 mM dithiothreitol, 40 mM chloroacetamide, Phosphatase Inhibitor Cocktail 2 (1:100, Sigma), Phosphatase Inhibitor Cocktail 3 (1:100, Sigma)) and cooked at 95°C for 10 min. The tissue lysates were incubated for 30 min with Benzonase to degrade DNA and RNA followed by overnight digestion of proteins by Trypsin and LysC at 37°C. The samples were acidified by adding formic acid to reach a final concentration of 1% to stop the digestion and precipitate the SDC. The tissue debris and SDC were removed by centrifugation and the peptide-containing supernatants were desalted on a SepPak C18 column (Waters) following the manufacturer's instructions. The peptide concentrations were determined using a Pierce BCA Protein Assay (Thermo Scientific), the samples dried in a vacuum concentrator and were stored at -80°C until measurement.

LC–MS/MS analysis: Lyophilized peptides were reconstituted in MS sample buffer (3% ACN/0.1% FA) and 1 µg peptide was injected into the LC-MS/MS system consisting of an Vanquish Neo UHPLC 2109 (Thermo Fisher Scientific) coupled to an orbitrap Exploris 480 mass spectrometer (Thermo Fisher Scientific). Samples were separated on an in-house packed 20 cm × 75 µm ID analytical column with 1.9 µm C18 ReproSil beads with a gradient of sample buffer and buffer B (90% ACN/0.1% FA). Buffer B was increased from 4% to 20% in the first 68 min, followed by an increase to 30% B in 20 min and another increase to 60% B in 10 min. MS data were acquired in data-independent acquisition mode (DIA). Full scans were conducted at 120K resolution with normalized AGC target of 300%, mass range of 350-1650 m/z and a maximum injection time of 20 ms. Each full scan was followed by MS2 scans with a window width of 12 m/z, at 30 K resolution, with a normalized AGC target of 1000%, stepped normalized collision energy (26, 29, 32) and a maximum injection time of 54 ms.

Proteomic data preprocessing and statistical analysis: The raw data were analyzed using directDIA in Spectronaut version 19.5. Standard settings were adjusted by reducing the run-wise protein Q value to 0.01 and adding Deamidation (NQ) as a variable modification. The spectra were searched against a mouse Uniprot database from 05/2024 including isoforms. Further downstream analysis was performed in R (version 4.4.3).

Raw protein group intensities were log2-transformed and assessed for data completeness. Samples were evaluated for the number of identified proteins, and potential outliers were flagged using the interquartile range (IQR) method applied to protein count distributions. Based on this assessment, one RV sample with markedly low protein coverage was excluded from downstream analysis. Protein groups supported

by fewer than two peptides were removed. To minimize the influence of missing values, we required proteins to be quantified in at least 80% of samples within at least one experimental group (i.e., LV or RV tissue from chow, HFpEF, Hx, or RV-HFpEF mice). Remaining missing values were imputed from a normal distribution (width = 0.3, shift = 1.8, log2 scale), simulating low-abundance proteins. Differential expression analysis was performed using the limma package[90] (3.62.2) in R. A linear model was constructed including group and chamber information, while accounting for biological replicates using duplicate correlation. Pairwise contrasts were defined for all biologically relevant comparisons between treatment groups and chambers. Moderated t-statistics were computed with empirical Bayes shrinkage, and differentially expressed proteins were defined as those with Benjamini–Hochberg adjusted $p < 0.05$ and absolute fold change >1.5.

A preranked gene set enrichment analysis (GSEA) was performed using the fgsea package[91] (version 1.32.4) in R. Proteins were ranked according to the moderated t-statistics obtained from the limma analysis. Where multiple protein entries mapped to the same gene symbol, the entry with the largest logFC in the RV-HFpEF vs. chow contrast was kept. Gene symbols were used to generate ranked gene lists for each contrast.

Pathway annotations (Reactome and Gene Ontology (GO) Biological Pathways version 2026.1) were obtained from the Molecular Signatures Database (MSigDB). Pre-ranked GSEA was performed separately for each contrast and gene set database using the fgsea algorithm with pathway size limits of 10–500 genes. Normalized enrichment scores (NES), adjusted p-values (Benjamini–Hochberg false discovery rate correction), and leading-edge genes were extracted.

For visualization, the ten most positively enriched and ten most negatively enriched pathways in the RV-HFpEF vs. chow comparison were selected based on NES. Pathway enrichment across contrasts was summarized using heatmaps displaying NES values. Pathways with an adjusted p-value < 0.05 were indicated by an asterisk. The top pathways in the RV-HFpEF vs. chow comparison were also visualized using bubble plots (normalized enrichment score vs. pathway, point size proportional to the number of contributing proteins, and point color representing overlap percentage).

To visualize protein expression patterns within selected Reactome pathways and GO terms (“inflammatory response”, “innate immune system”, and “extracellular matrix organization”), proteins with a log2 fold change > 1 and an FDR < 0.05 in the RV-HFpEF versus chow comparison were first identified. From this set, proteins assigned to the respective gene sets in MSigDB and quantified in all samples (i.e., without missing values) were selected. Normalized protein intensities were transformed to z-scores across samples and visualized as heat maps. Protein selection was therefore based on differential expression (log2 fold change > 1, FDR < 0.05), pathway membership, and data completeness.

Echocardiography

Image acquisition was performed using an ultrahigh frequency linear array transducer (MX400, 20–46 MHz) in combination with a Vevo® 3100 high-resolution imaging system (FUJIFILM VisualSonics, Toronto, ON, Canada). Mice were initially anesthetized with 3–4% isoflurane, which was reduced to 1.5%–2% to maintain anesthesia and were placed in supine position on a heating pad to maintain body temperature and heart rates at physiological and comparable ranges. ECGs were monitored (FUJIFILM VisualSonics) to ensure heart rates were comparable between groups. Pre-warmed ultrasound gel (Parker Laboratories Fairfield, New Jersey, USA) was applied on the depilated chest to obtain brightness (B)- and motion (M)-Mode images from parasternal long axis views of the LV to assess systolic and diastolic function and dimensions. Modified parasternal short axis images of the pulmonary artery trunk were obtained by B-mode shifting. We applied pulsed-wave and color Doppler settings prior to the bifurcation of the right and left pulmonary arteries to measure peak pulmonary artery flow profiles. LV

tissue velocity profiles were taken at the septal mitral annulus with tissue Doppler in an apical four chamber view orientation. Transmitral flow patterns were measured with pulsed-wave Doppler using color doppler guidance to identify the maximal flow rates in an apical four chamber view. M-mode images for illustration of the systolic longitudinal displacement of the lateral tricuspid annulus towards the apex were measured in an apical four-chamber view orientation. Images were stored as raw data in DICOM format for offline analysis.

Analysis of echocardiographic images

Image analysis was performed using the VevoLAB Version 5.7.1 (FUJIFILM VisualSonics). Ejection fraction, stroke volume, end-diastolic volume and heart rate were calculated from B-Mode images of the LV in a parasternal long axis view using the LV Trace tool. End-diastolic diameter was measured from M-mode images of the LV in a parasternal long axis view using the semi-automatic LV Trace tool. Two-dimensional speckle-tracking echocardiography to assess global longitudinal peak strain (GLS) was performed using VevoStrain software (FUJIFILM VisualSonics) and a speckle-tracking algorithm. For each animal, semi-automated endocardial border tracing was applied by pre-selecting three consecutive cardiac cycles of a B-Mode cine loop acquired from the parasternal long-axis view in end-systole. B-mode images were selected based on frame rates > 200 fps and on visibility of endocardial and epicardial LV border regions. Peak global longitudinal strain values were calculated from six independent anatomical segments of the LV.

Invasive hemodynamics

RV pressure profiles were recorded by invasive hemodynamic monitoring and served as the primary outcome measure. Mice were anaesthetized intraperitoneally (i.p.) with ketamine (120 µg/g body weight) and xylazine (16 µg/g body weight), and anesthetic depth was regularly checked by the toe pinch reflex. Animals were placed in a supine position on a heating pad to maintain body temperature which was regularly controlled via a rectal probe during the procedure. After microsurgically exposing the external jugular vein, a 1F microtip pressure catheter (Millar, Houston, Tx) was inserted into the trifurcation of the axillary vein, external jugular vein and anterior jugular vein with the subclavian vein before it was advanced towards the RV. Stable pressure profiles of the RV were recorded for at least 2 minutes by PowerLab 4/35 connected to LabChart v. 8 software (ADInstruments, Sydney, Australia). After completion of the measurements, animals were euthanized by cervical dislocation.

Analyses of invasive hemodynamics

The recorded RV pressure data were analyzed using LabChart v. 8 software (ADInstruments, Sydney, Australia) by a blinded observer. We measured the difference between maximum and minimum ventricular pressures in 10 seconds periods in stable pressure curves (ΔP). The average ΔP was calculated to give the RVSP.

Treadmill experiments

Mice were initially acclimatized to the treadmill (CaloTreadmill, TSE Systems, Germany) by running uphill (20°) at a constant speed (5 m/min) for 5 min for 3 consecutive days. After acclimatization, an exhaustion test was performed by running uphill (20°) on the treadmill at warm-up speed (5 m/min) for 4 min and then increasing the speed to 14 m/min for 2 min. Every 2 minutes thereafter, the speed was increased by 2 m/min until the animal was exhausted. Exhaustion was defined by three separate criteria:

(i) the animal is exposed to the shock grid for 5 seconds or more, (ii) the animal is exposed to the shock grid three times in a row for less than 10 seconds with 15 seconds in between, or (iii) the animal spent less than 2 s on the treadmill before contacting the shock grid again (i.e. lack of motivation). Running distances were automatically collected and processed with PhenoMaster software V7.1.6 (TSE Systems, Germany).

Histology

For fibrosis staining RV and LV tissue preparations were stored in 4 % paraffin (SAV Liquid Production GmbH) followed by storage in 1x PBS. Dehydration was performed by immersion in increasing ethanol-series (70 %, 80 %, 96 %, 2X 100 %) completed with incubation in paraffin for 1h each step. After embedding in paraffin 5 µm thick sections were prepared, dried at 65°C and deparaffinized through Neo-Clear (2X 5 min, Sigma-Aldrich) and decreasing alcohol series (2X 100 %, 96 %, 80 %, 70 %, 5 min each step) finished with distilled water (2X 5 min). The slides were stained with Picro-Sirius Red Solution (Abcam) for 30 min followed by incubation in acetic acid (2X 5 min, 0,5 %, Roth). For tissue dehydration the slides were immersed through a series of increasing ethanol concentrations (6 dips, 96 %, 3X 100 %). After incubation in Neo-Clear (2X 5 min) the slides were mounted in Neo-Mount (Sigma-Aldrich) and dried overnight.

Analyses of histology

For imaging, Nano Zoomer (Hamamatsu) equipped with a 40x objective was used for fine focus. Analysis was performed using NDP.scan SQ 1.0 Imaging Software. Total collagen levels of 5-10 different areas per heart cross-section were calculated by dividing the red-stained collagen area by total tissue area using ImageJ.

Re-analyses of bulk RNA-sequencing data

HTSeq-count and DESeq2 were used to obtain normalized gene counts for each subject (n=41 HFpEF and n=24 controls using previously published bulk RNA-seq data from RV septal tissue[24]. Gene expression was normalized using the DESeq2 median of ratios normalization. Differential expression between HFpEF vs controls was performed using DESeq2. P-values were adjusted using the Benjamini-Hochberg method. Violin plots display the normalized expression for each group (HFpEF or controls) and adjusted p-values from differential expression testing performed with DESeq2.

Supplemental Figures

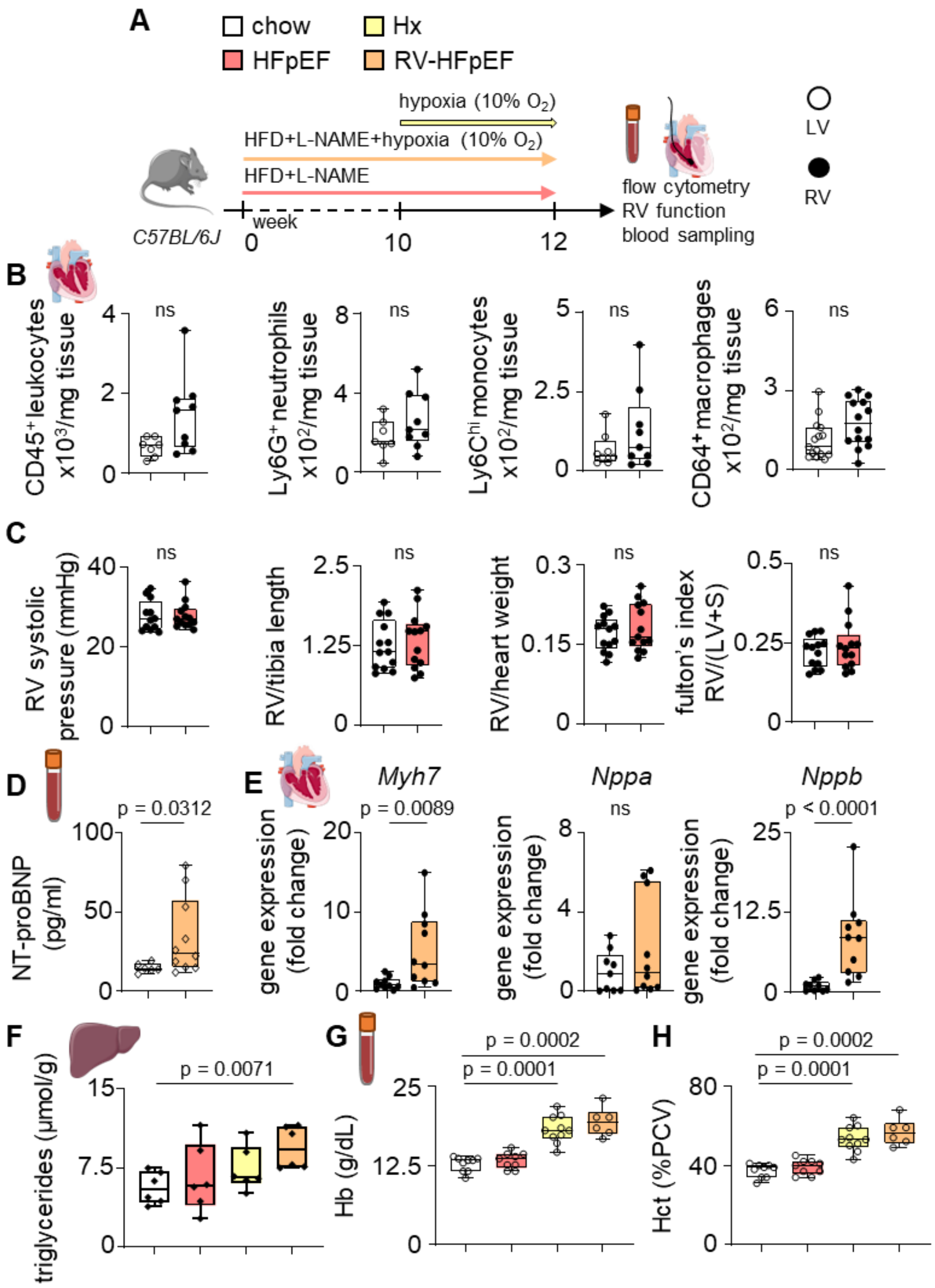

Figure S1 | The two-hit HFpEF model does not develop RV dysfunction.
A, Experimental outline of *C57BL/6J* mice treated with i) chow, ii) HFpEF (L-NAME, 60% HFD), iii) chronic hypoxia (Hx, 10% O_2) or iv) HFpEF and hypoxia (RV-HFpEF). **B,** Flow cytometric assessed LV and RV leukocyte (n=7-9), neutrophil (n=7-9), monocyte (n=7-9) and macrophage (n=13-15) counts in chow mice. **C,** RV systolic pressure from chow and HFpEF (n=12-13) mice. RV weight-to-tibia length ratio (RV/tibia length), RV weight-to-heart weight ratio (RV/heart weight) and fulton's index (RV mass relative to LV and septal mass (RV/(LV+S)) from chow and HFpEF mice (n=13). **D**, NT-proBNP measurements from chow and RV-HFpEF mice (n=6-10). **E**, *Myh7*, *Nppa* and *Nppb* relative gene expression in RV bulk tissue of chow and RV-HFpEF (n=9-10). Expression values were calculated relative to *Sdha* expression using the $2^{-\Delta\Delta Ct}$ method. Mann–Whitney test was used. **F**, Hepatic triglyceride levels in chow, HFpEF, Hx and RV-HFpEF mice (n=6). **G**, Hb and **H**, Hct levels in chow, HFpEF, Hx and RV-HFpEF (n=6-10) mice. P values were calculated using a Mann–Whitney test (B-D), or a Kruskal-Wallis test followed by uncorrected Dunn's multiple comparisons test (F-H). Data are presented as median with interquartile range (IQR). HFpEF, heart failure with preserved ejection fraction. LV, left ventricle, RV, right ventricle. HFD, high-fat diet. L-NAME, Nω-nitro-L-arginine methylester hydrochloride. Hb, hemoglobin. Hct, hematocrit. ns, not significant.

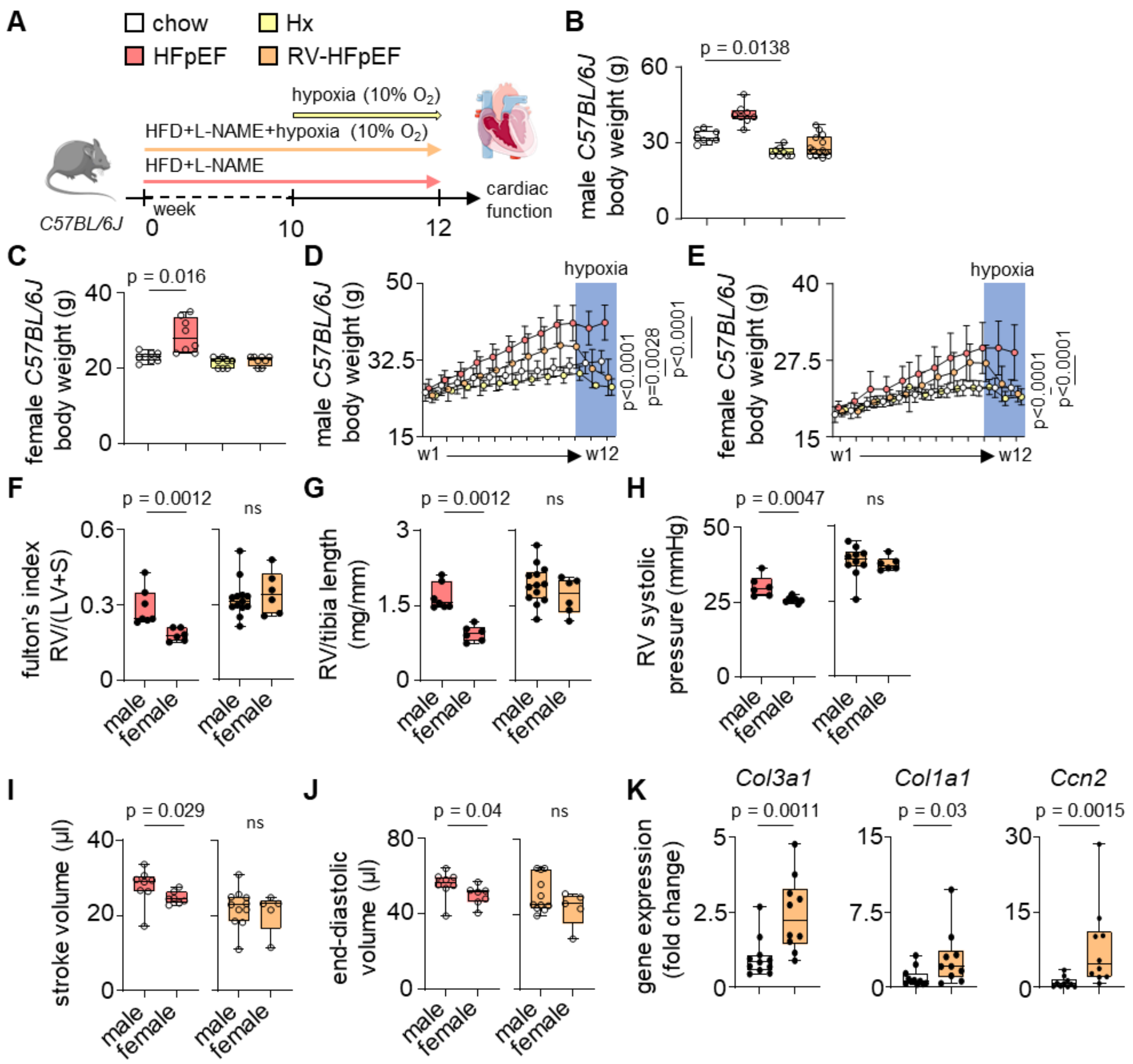

Figure S2 | Chronic hypoxia treatment reduces sex differences in ventricular function observed in the two-hit HFpEF model.

A, Experimental outline of *C57BL/6J* mice treated with i) chow, ii) HFpEF (L-NAME, 60% HFD), iii) chronic hypoxia (Hx, 10% O_2) or iv) HFpEF and hypoxia (RV-HFpEF). **B,** Body weights from male (n=8-14 per group) and **C**, female mice (n=8 per group) after 12 weeks. **D,** Body weight changes over time of male (n=8-14 per group) and **E**, female mice (n=8 per group) over 12 weeks of RV-HFpEF. **F**, Comparison of fulton's index (RV mass relative to LV and septal mass (RV/(LV+S)), n=6-13), **G**, RV weight-to-tibia length ratio (RV/tibia length, n=6-13), **H**, RV systolic pressure (n=6-10), **I**, stroke volume (n=5-11) and **J**, end-diastolic left ventricular volumes (n=5-11) of male and female HFpEF and RV-HFpEF mice. **K,** *Col3a1*, *Col1a1* and *Ccn2* relative gene expression in RV bulk tissue of chow and RV-HFpEF mice (n=10-11 per group). Expression values were calculated relative to *Sdha* expression using the $2^{-\Delta\Delta Ct}$ method. P values were calculated using a Kruskal-Wallis test followed by uncorrected Dunn's multiple comparisons test (B, C), a two-way ANOVA followed by uncorrected Fisher's least significant difference test (D,E), or a Mann–Whitney test (F,K). Data are presented as median with interquartile range (IQR). HFpEF, heart failure with preserved ejection fraction. RV, right ventricle. HFD, high-fat diet. L-NAME, Nω-nitro-L-arginine methylester hydrochloride. ns, not significant.

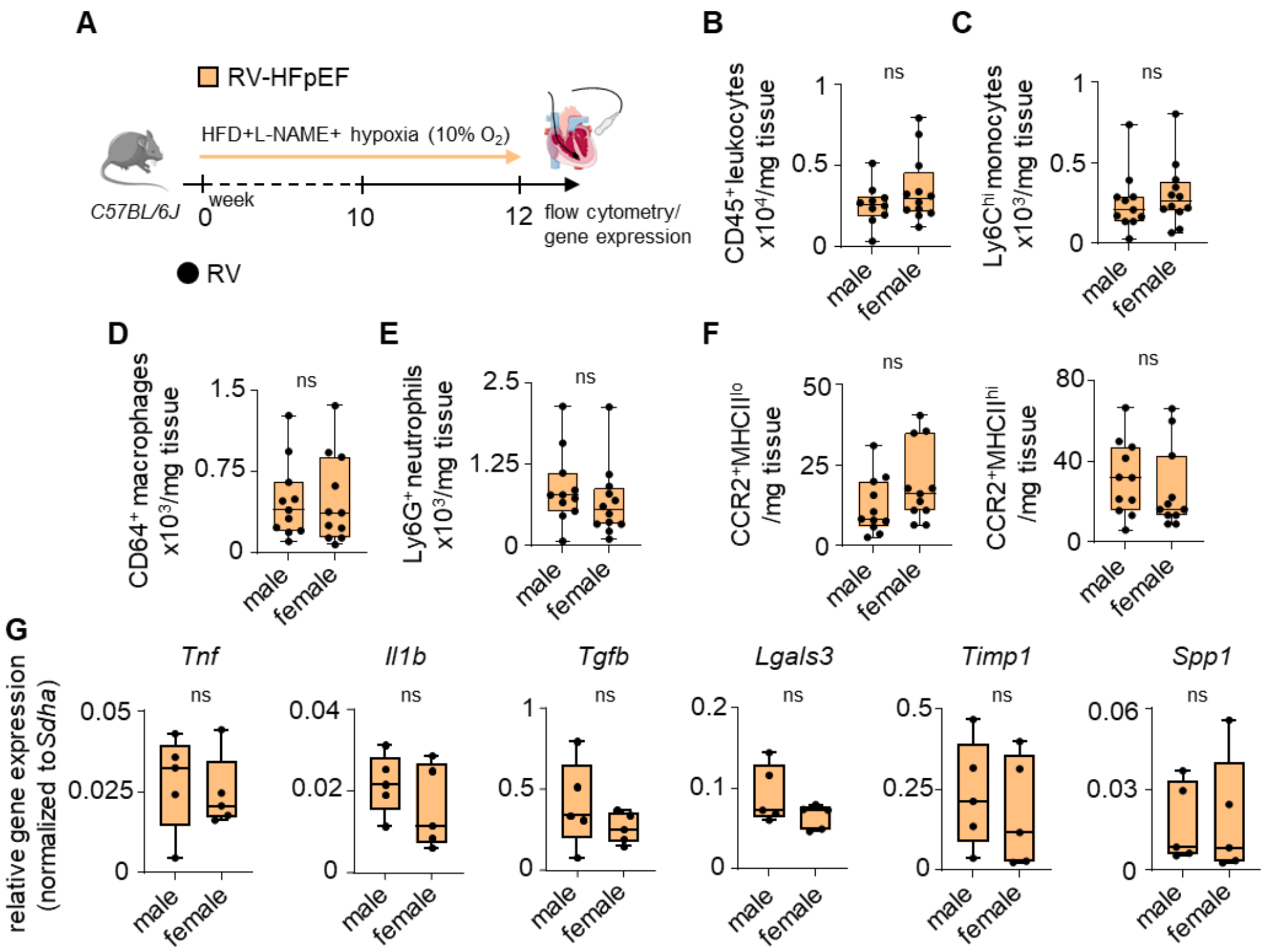

Figure S3 | RV immune cell accumulation and inflammation are independent of sex in RV-HFpEF mice.

A, Experimental outline of *C57BL/6J* mice treated with HFpEF (L-NAME, 60% HFD) and hypoxia (10% O_2) (RV-HFpEF). **B**, Flow cytometric quantification of $CD45^+$ leukocytes (n=10-12), **C**, $Ly6C^{hi}$ monocytes (n=11-12), **D**, $CD64^+$ macrophages (n=11). **E**, $Ly6G^+$ neutrophils (n=11-12) and **F**, $CCR2^+MHCII^{lo}$ (n=11) and $CCR2^+MHCII^{hi}$ (n=11) subsets in RV bulk tissue of male vs. female RV-HFpEF mice. **G**, Comparison of male vs. female *Tnf*, *Il1b*, *Tgfb*, *Lgals3*, *Timp1* and *Spp1* (n=5) relative gene expression in RV bulk tissue of RV-HFpEF mice. Gene expression was normalized to *Sdha* using the $2^{-\Delta Ct}$ method. P values were calculated using a Mann–Whitney test (B-G). Data are presented as median with interquartile range (IQR). HFpEF, heart failure with preserved ejection fraction. LV, left ventricle. RV, right ventricle. HFD, high-fat diet. L-NAME, Nω-nitro-L-arginine methylester hydrochloride. ns=not significant

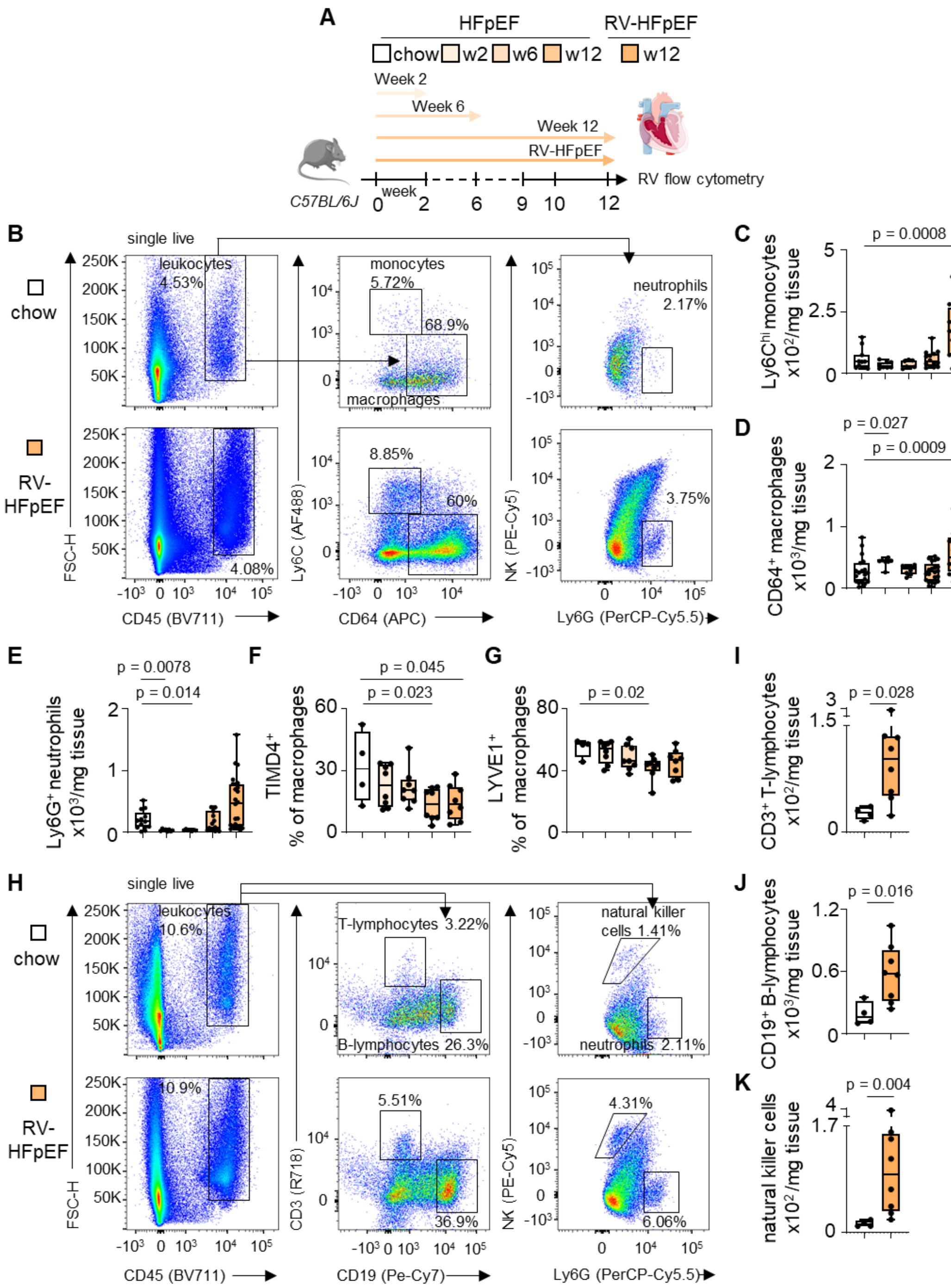

Figure S4 | Cardiac leukocyte subset dynamics in RV-HFpEF mice.
A, Experimental outline of longitudinal tracking of myeloid cell dynamics in *C57BL/6J* mice treated with i) chow, ii) 2 weeks of HFpEF (L-NAME, 60% HFD), iii) 6 weeks of HFpEF, iv) 12 weeks of HFpEF or v) HFpEF and chronic hypoxia (10% O_2, RV-HFpEF). **B**, Representative flow plots of RV myeloid cells in chow and RV-HFpEF mice. **C**, Flow cytometric quantification of RV Ly6C^{hi} monocytes (n=7-23), **D**, CD64^{+} macrophages (n=7-25), **E**, Ly6G^{+} neutrophils (n=7-24), **F**, TIMD4^{+} macrophages (n=4-8), and **G**, LYVE1^{+} macrophages of chow, HFpEF w2, HFpEF w6, HFpEF w12 and RV-HFpEF (n=4-8) mice. **H**, Representative flow plots of RV lymphocytes in chow and RV-HFpEF mice. **I**, Flow cytometric quantification of RV T-lymphocytes, **J**, B-lymphocytes and **K**, Natural killer cells of chow and RV-HFpEF (n=4-8). P values were calculated using a Kruskal-Wallis test followed by uncorrected Dunn's multiple comparisons test (C-G), or a Mann–Whitney test (I-K). Data are presented as median with interquartile range (IQR). W, week. HFpEF, heart failure with preserved ejection fraction. RV, right ventricle.

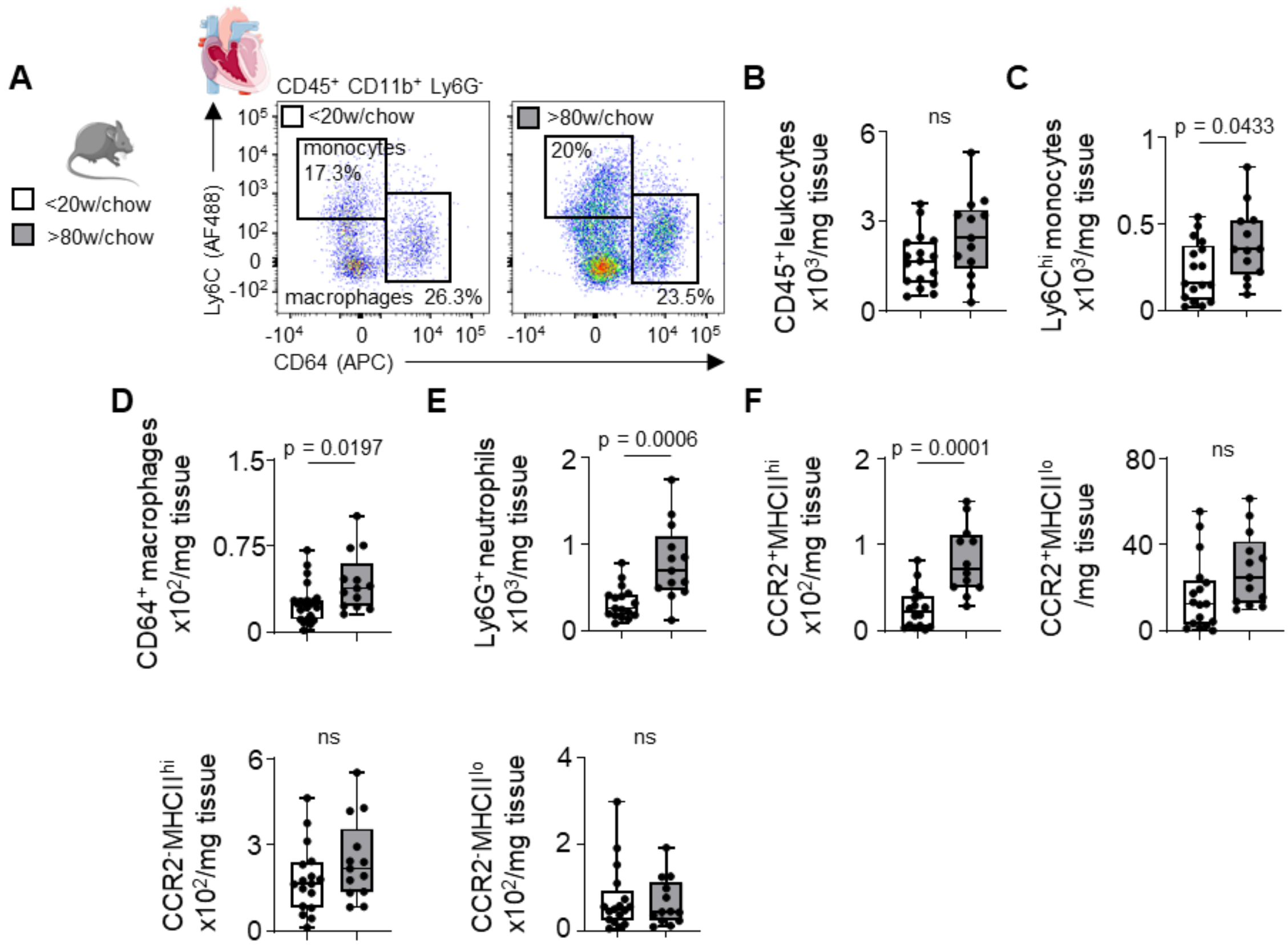

Figure S5 | Right ventricular myeloid cell expansion in aged mice.
A, Experimental outline of *C57BL/6J* mice at different biological ages (<20 weeks: young or >80 weeks: old, both chow diet) and representative flow plots. **B**, Flow cytometric quantification of RV $CD45^+$ leukocytes (n=13-17), **C**, $Ly6C^{hi}$ monocytes (n=13-17), **D**, $CD64^+$ macrophages (n=13-22), **E**, $Ly6G^+$ neutrophils (n=13-22) and **F**, $CCR2^+MHCII^{hi}$ (n=12-16), $CCR2^+MHCII^{lo}$ (n=13-17), $CCR2^-MHCII^{hi}$ (n=13-17) and $CCR2^-MHCII^{lo}$ subsets (n=13-17). P values were calculated using a Mann–Whitney test (B-F). Data are presented as median with interquartile range (IQR). ns=not significant.

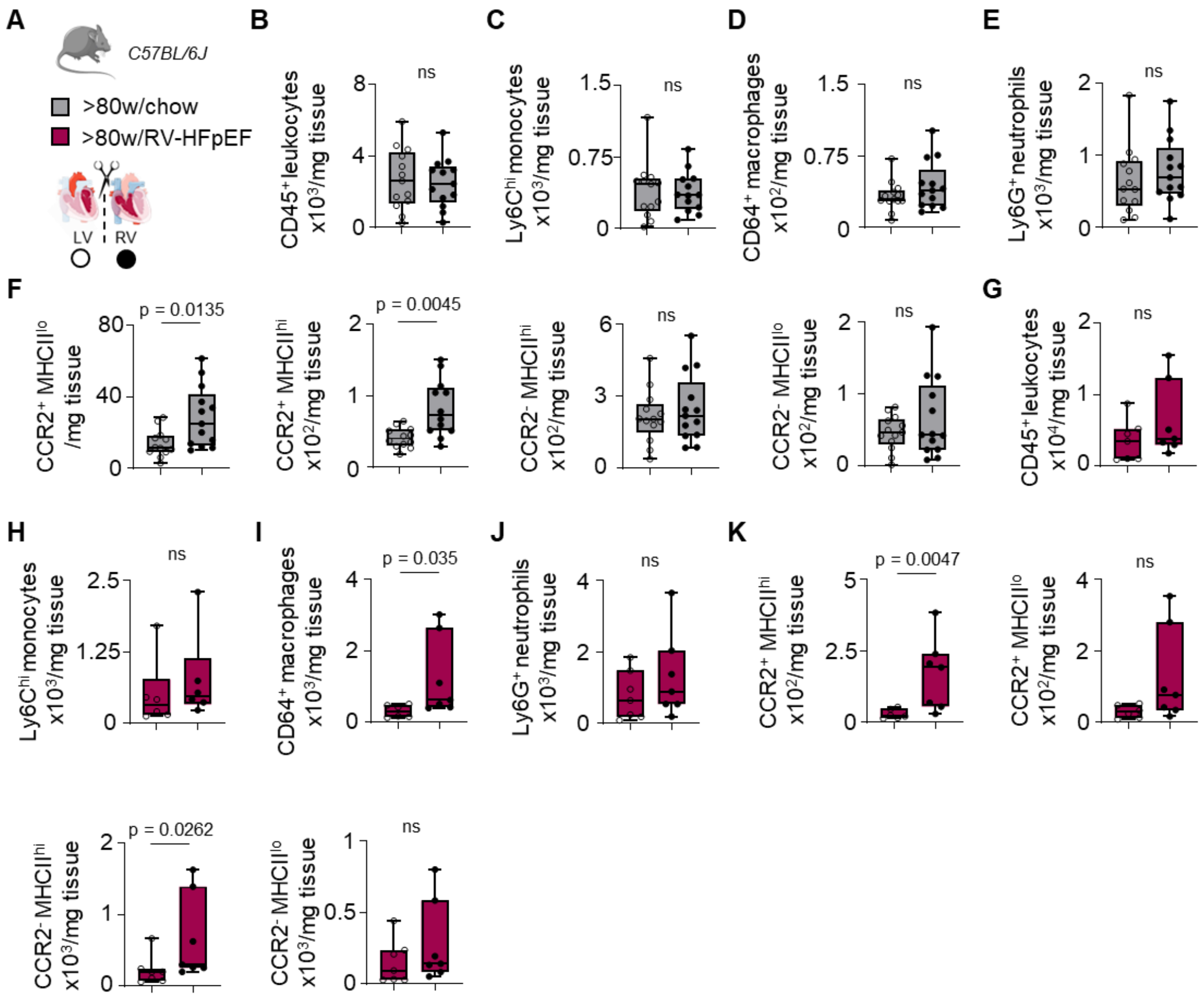

Figure S6 | Age is associated with increased immune cell accumulation in RV tissue.
A, Experimental outline of *C57BL/6J* mice treated with chow (>80w) or HFpEF (L-NAME, 60% HFD) and hypoxia (10% O_2, RV-HFpEF, >80w). **B**, Comparison of LV and RV CD45$^+$ leukocytes (n=13), **C**, Ly6C^{hi} monocytes (n=13), **D**, CD64$^+$ macrophages (n=13), **E**, Ly6G$^+$ neutrophils (n=13) and **F**, CCR2$^+$MHCII^{lo} (n=12-13), CCR2$^+$MHCII^{hi} (n=12), CCR2$^-$MHCII^{hi} (n=13) and CCR2$^-$MHCII^{lo} subset (n=13) in chow/>80w mice. **G**, Comparison of LV and RV CD45$^+$ leukocytes (n=7), **H**, Ly6C^{hi} monocytes (n=6), **I**, CD64$^+$ macrophages (n=6-7), **J**, Ly6G$^+$ neutrophils (n=7) and **K,** CCR2$^+$MHCII^{hi} (n=6-7), CCR2$^+$MHCII^{lo} (n=6-7), CCR2$^-$MHCII^{hi} (n=7) and CCR2$^-$MHCII^{lo} (n=7) subsets in RV-HFpEF/>80w mice. P values were calculated using a Mann–Whitney test (B-K). Data are presented as median with interquartile range (IQR). HFpEF, heart failure with preserved ejection fraction. LV, left ventricle. RV, right ventricle. HFD, high-fat diet. L-NAME, Nω-nitro-L-arginine methylester hydrochloride. ns, not significant.

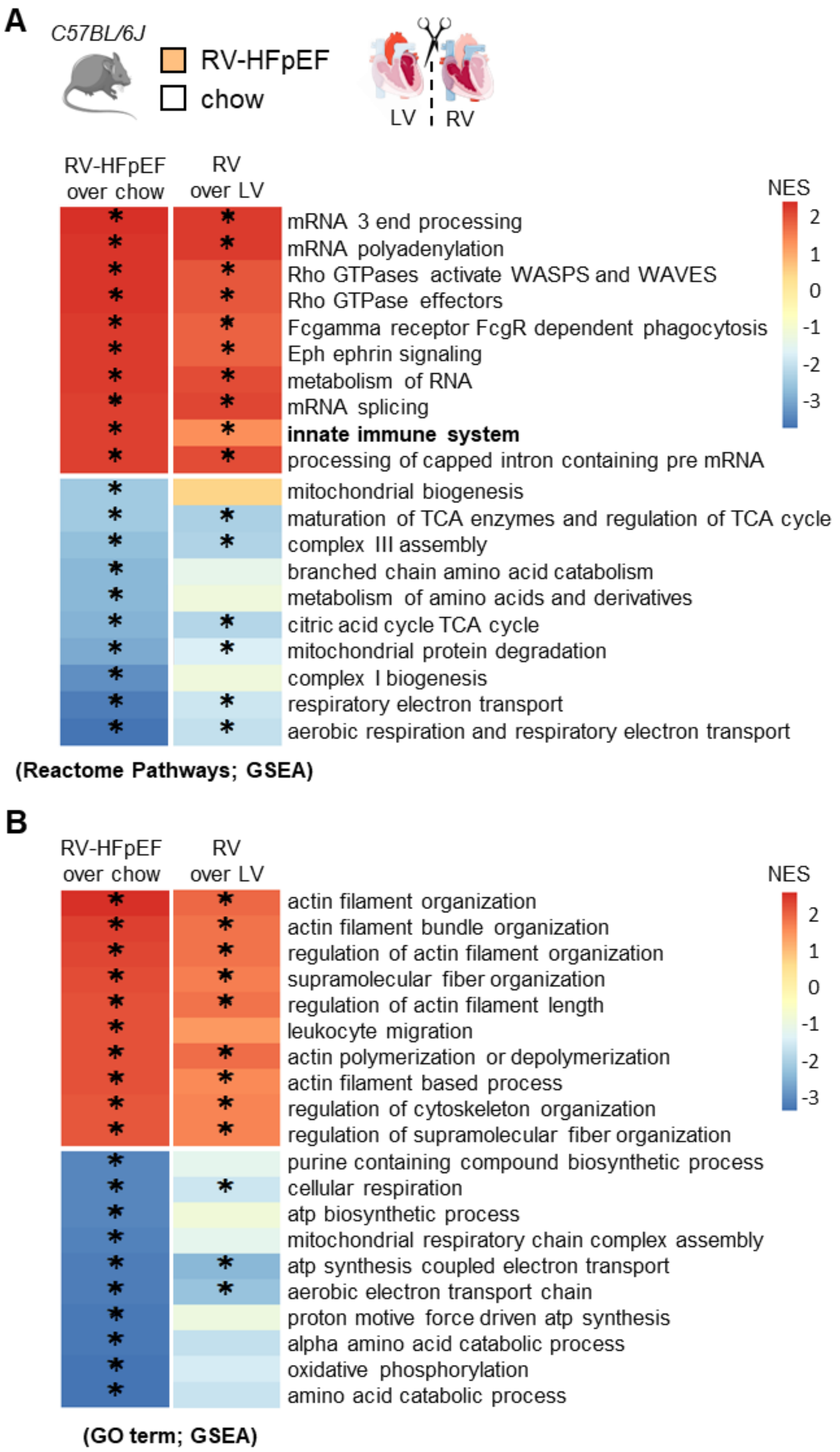

Figure S7 | Immune system activation in RV bulk tissue of RV-HFpEF mice.
A, Heatmap showing the 10 most positively and 10 most negatively enriched Reactome pathways and **B**, Gene Ontology Biological Process (GO) terms by Gene Set Enrichment Analysis (GSEA). The terms most different based on the RV-HFpEF versus chow comparison were chosen and the normalized enrichment scores (NES) of these pathways are shown for the RV versus LV comparison in RV-HFpEF mice. NES were calculated using a weighted Kolmogorov–Smirnov-like running-sum statistic based on proteins ranked by moderated t-statistics. Red and blue indicate positive and negative enrichment, respectively. Asterisks denote significantly enriched pathways or GO terms (FDR < 0.05). The pathway marked in bold ("innate immune system") confirms our initial findings from flow cytometry (leukocyte expansion in RV-HFpEF RVs). HFpEF, heart failure with preserved ejection fraction. RV, right ventricle. LV, left ventricle. NES, Normalized Enrichment Score.

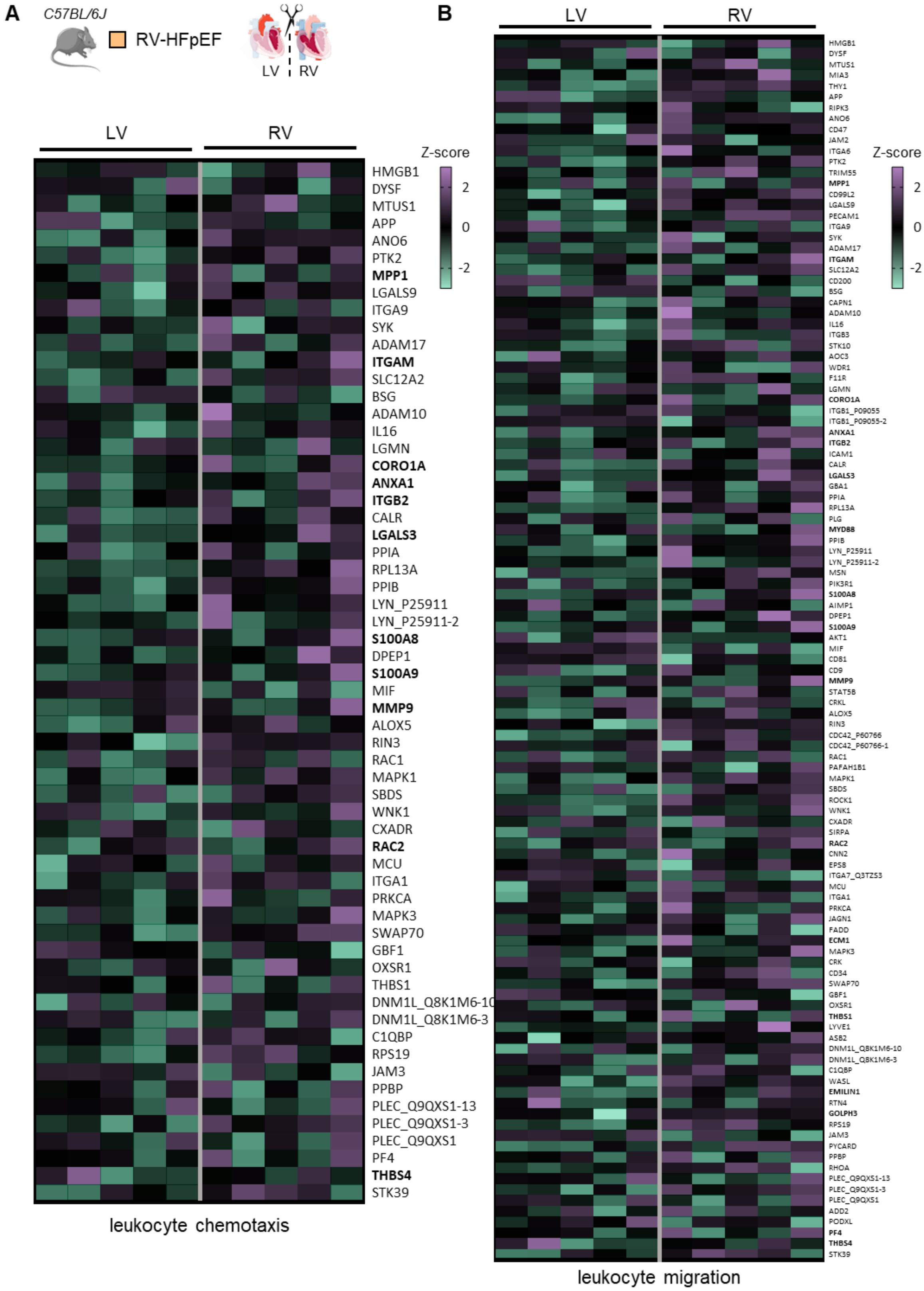

Figure S8 | Proteins associated with leukocyte chemotaxis and migration are enriched in RV tissue of RV-HFpEF mice.

A, **B,** Heatmap of protein expression values (z-scored) in RV and LV tissue of RV-HFpEF mice (n=5) annotated with the GO term "leukocyte chemotaxis" (**A**) and "leukocyte migration" (**B**). Only proteins that have been measured in all animals and both ventricles are illustrated. The proteins marked in bold are proteins with a log2 fold change > 1 and an FDR < 0.05 in the RV-HFpEF versus chow comparison. HFpEF, heart failure with preserved ejection fraction. RV, right ventricle. LV, left ventricle.

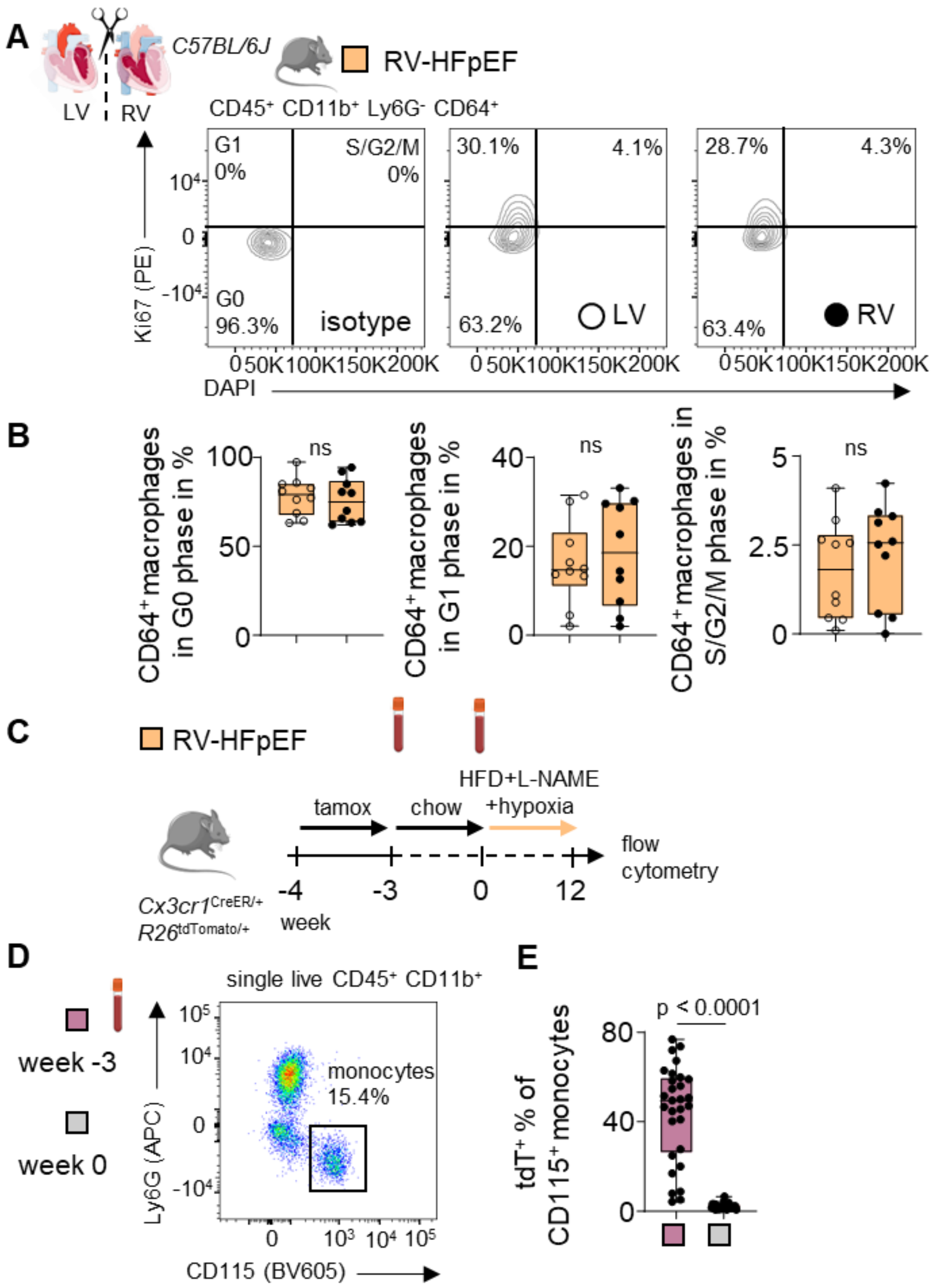

Figure S9 | Ventricular macrophages represent elevated proliferation rates in response to RV-HFpEF.

A, Representative flow plots of cell cycle analysis gated on $CD45^+$ $CD11b^+$ $Ly6G^-$ $CD64^+$ macrophages stained with Ki-67 antibody and DAPI in LVs and RVs from RV-HFpEF mice. The isotype control was used for proper gating. **B,** Percentage of LV and RV $CD64^+$ macrophages in G0 phase (left), G1 phase (mid) and S/G2/M phase (right, n=10 per group) of total macrophages. **C**, Experimental outline of Fate-mapping in $Cx3cr1^{CreER/+}R26^{tdTomato/+}$ RV-HFpEF mice treated with tamoxifen-rich diet (tamox) for 1 week prior to a 3-week wash out (chow) followed by HFpEF (L-NAME, 60% HFD) and chronic hypoxia (Hx, 10% O_2). **D**, Representative flow plot and **E**, Quantification of tdTomato (tdT) signal in blood $CD115^+$ monocytes after tamoxifen treatment (week -3) and tamoxifen wash out (week 0, n=29). P values were calculated using a Mann–Whitney test (B,D,E). Data are presented as median with interquartile range (IQR). HFpEF, heart failure with preserved ejection fraction. RV, right ventricle. LV, left ventricle. HFD, high-fat diet. L-NAME, Nω-nitro-L-arginine methylester hydrochloride. tamox, tamoxifen-rich diet. ns, not significant.

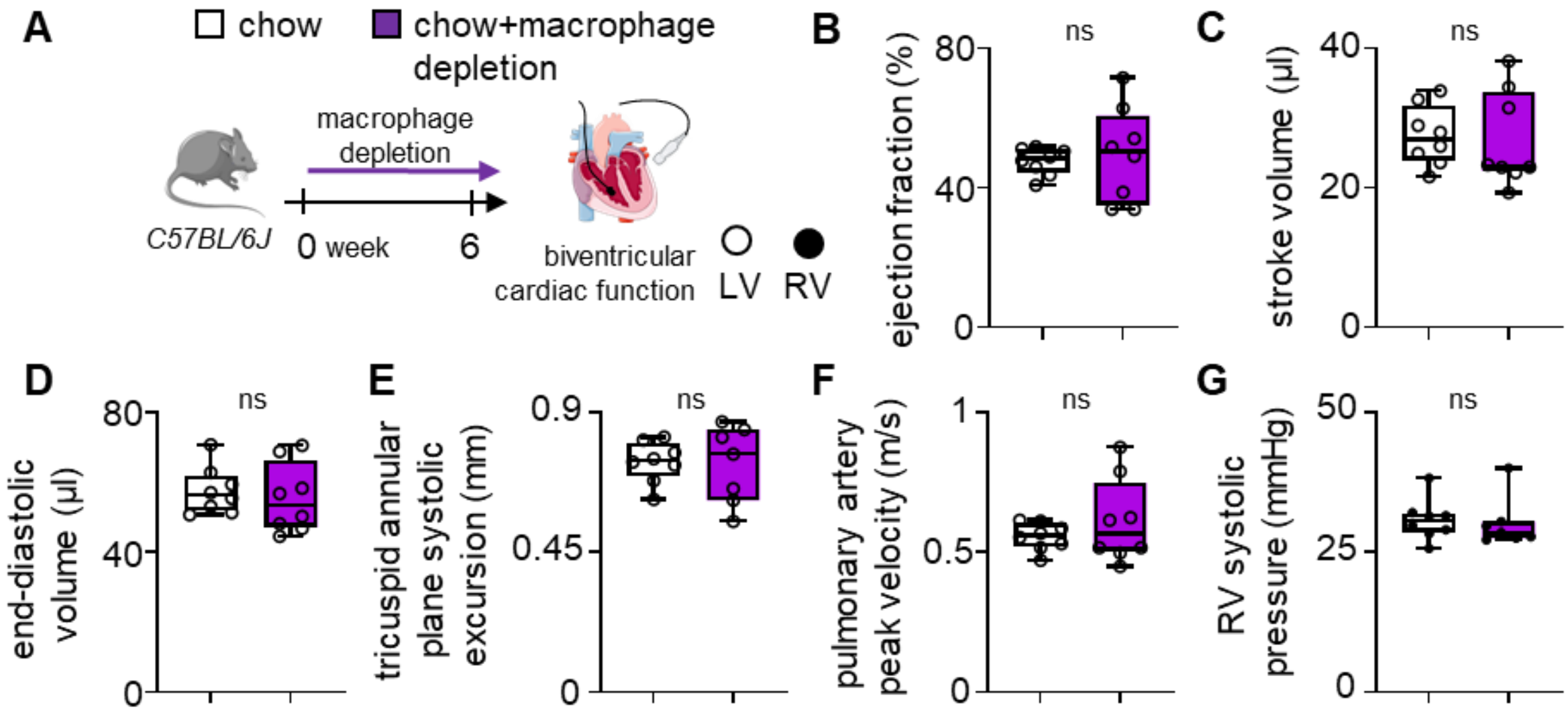

Figure S10 | Macrophage depletion in control mice does not affect LV function.

A, Experimental outline for chow mice treated with colony-stimulating factor 1 receptor (CSF1R) inhibitor (PLX5622) for 6 weeks. **B**, Echocardiographic assessment of ejection fraction, **C**, stroke volume, **D**, end-diastolic left ventricular volume, **E**, TAPSE and **F**, pulmonary artery peak velocity profiles in controls and mice fed with PLX5622 (each n=7-8). **G**, Quantitative analyses of RV systolic pressures in controls and mice fed with PLX5622 (n=7-8). P values were calculated using a Mann–Whitney test (B-G). Data are presented as median with interquartile range (IQR). RV, right ventricle. LV, left ventricle. TAPSE, tricuspid annular plane systolic excursion. ns, not significant.

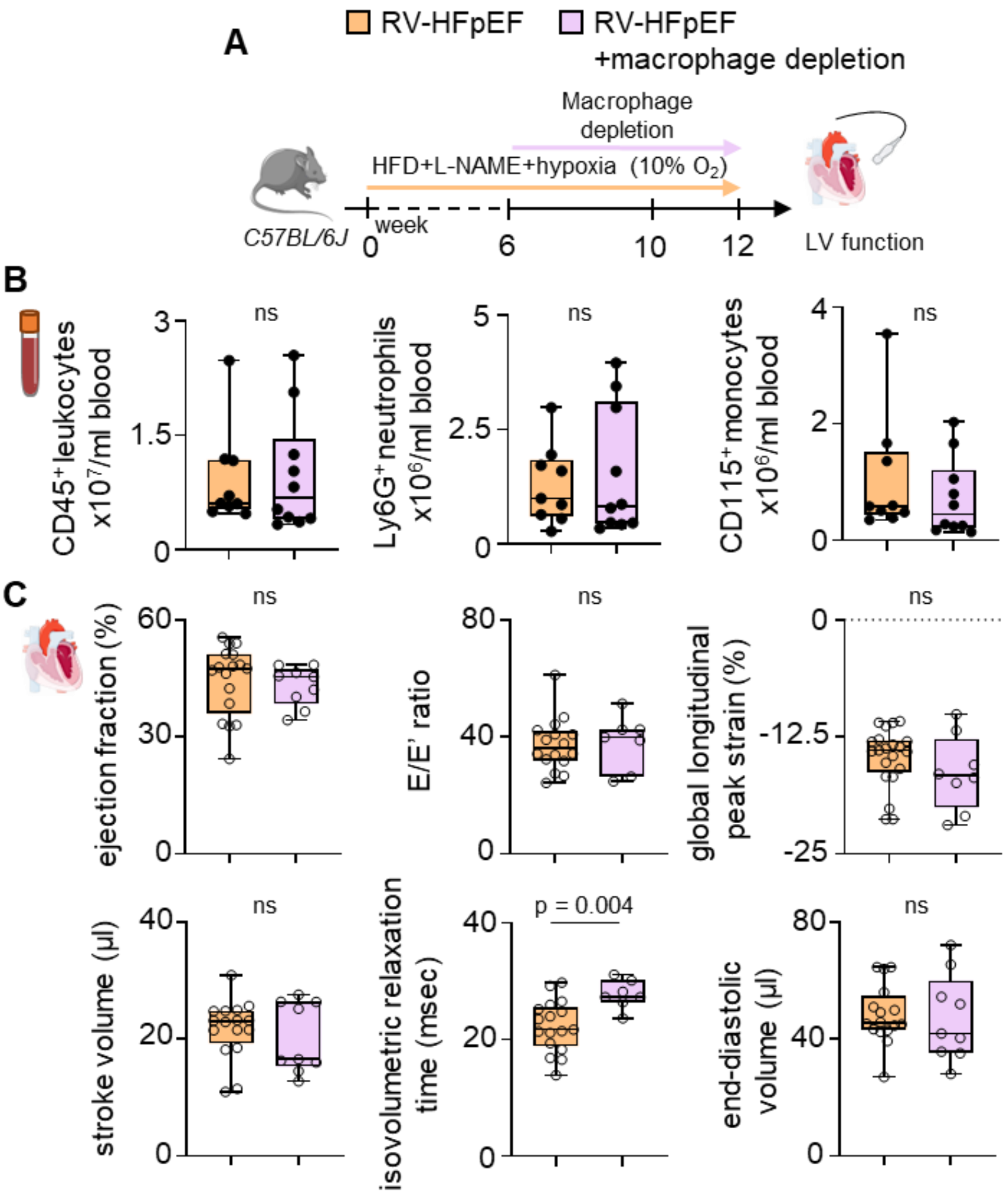

Figure S11 | Macrophage depletion in RV-HFpEF mice does not impact blood leukocyte levels or LV function.

A, Experimental outline for RV-HFpEF mice treated with colony-stimulating factor 1 receptor (CSF1R) inhibitor (PLX5622) for the last 6 weeks of the experiment (RV-HFpEF+PLX5622). **B,** Comparison of RV $CD45^{+}$ leukocyte, $Ly6G^{+}$ neutrophil and $CD115^{+}$ monocyte counts of RV-HFpEF and RV-HFpEF+PLX5622 (n=9-10 per group). **C,** Echocardiographic assessment of LV ejection fraction (n=9-17), mitral valve annular early diastolic velocity (E/E′, n=7-9), global longitudinal peak strain (n=8-21), stroke volume (n=9-16), isovolumetric relaxation time (n=7-17) and end-diastolic volume (n=9-16) of RV-HFpEF and RV-HFpEF+PLX5622 mice. P values were calculated using a Mann–Whitney test (B,C). Data are presented as median with interquartile range (IQR). HFpEF, heart failure with preserved ejection fraction. LV, left ventricle. RV, right ventricle. HFD, high-fat diet. L-NAME, Nω-nitro-L-arginine methylester hydrochloride. ns, not significant.

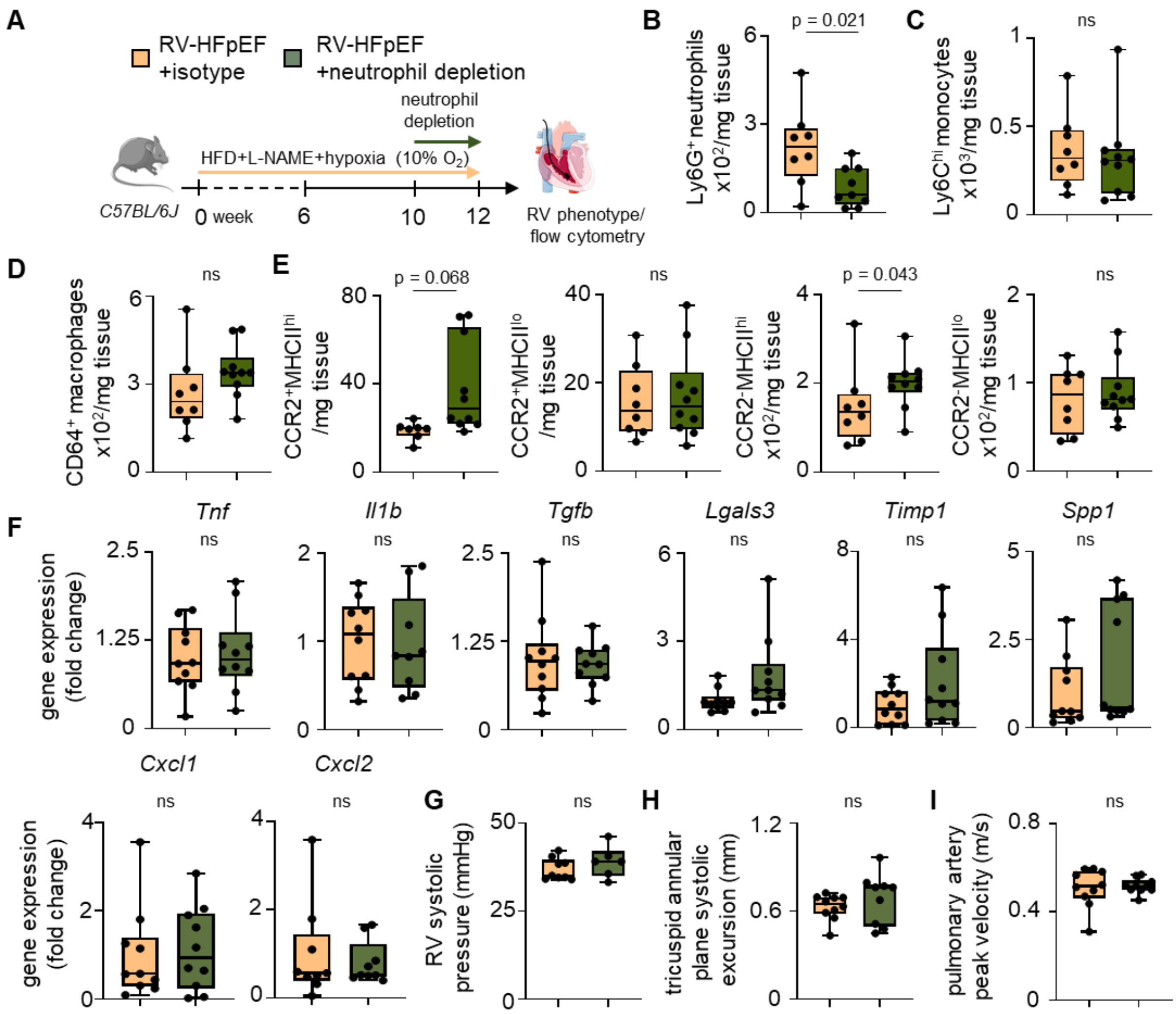

Figure S12 | Neutrophil depletion in RV-HFpEF mice does not improve RV function.

A, Experimental outline for RV-HFpEF mice treated with neutrophil depletion (RV-HFpEF+neutrophil depletion) or isotype control antibodies (RV-HFpEF+isotype) for the last 2 weeks of the experiment. **B**, Quantification of Ly6G^{+} neutrophils (n=8-10), **C**, Ly6C^{hi} monocytes (n=8-10), **D**, CD64^{+} macrophages (n=8-10), **E**, and macrophage subsets (n=8-10) in RV-HFpEF+isotype and RV-HFpEF+neutrophil depletion mice. **F**, *Tnf* (n=10), *Il1b* (n=9-10), *Tgfb* (n=10), *Lgals3* (n=10), *Timp1* (n=10), *Spp1* (n=10), *Cxcl1* (n=10) and *Cxcl2* (n=10) relative gene expression in RV bulk tissue of RV-HFpEF+isotype and RV-HFpEF+neutrophil depletion. Expression values were calculated relative to *Sdha* expression using the $2^{-\Delta\Delta Ct}$ method. **G**, Quantitative analysis of RV systolic pressure, **H**, TAPSE and **I**, pulmonary artery peak velocity profiles in RV-HFpEF+isotype and RV-HFpEF+neutrophil depletion (n=9-10) mice. P values were calculated using a Mann–Whitney test (B-I). Data are presented as median with interquartile range (IQR). HFpEF, heart failure with preserved ejection fraction. RV, right ventricle. TAPSE, tricuspid annular plane systolic excursion. ns, not significant.

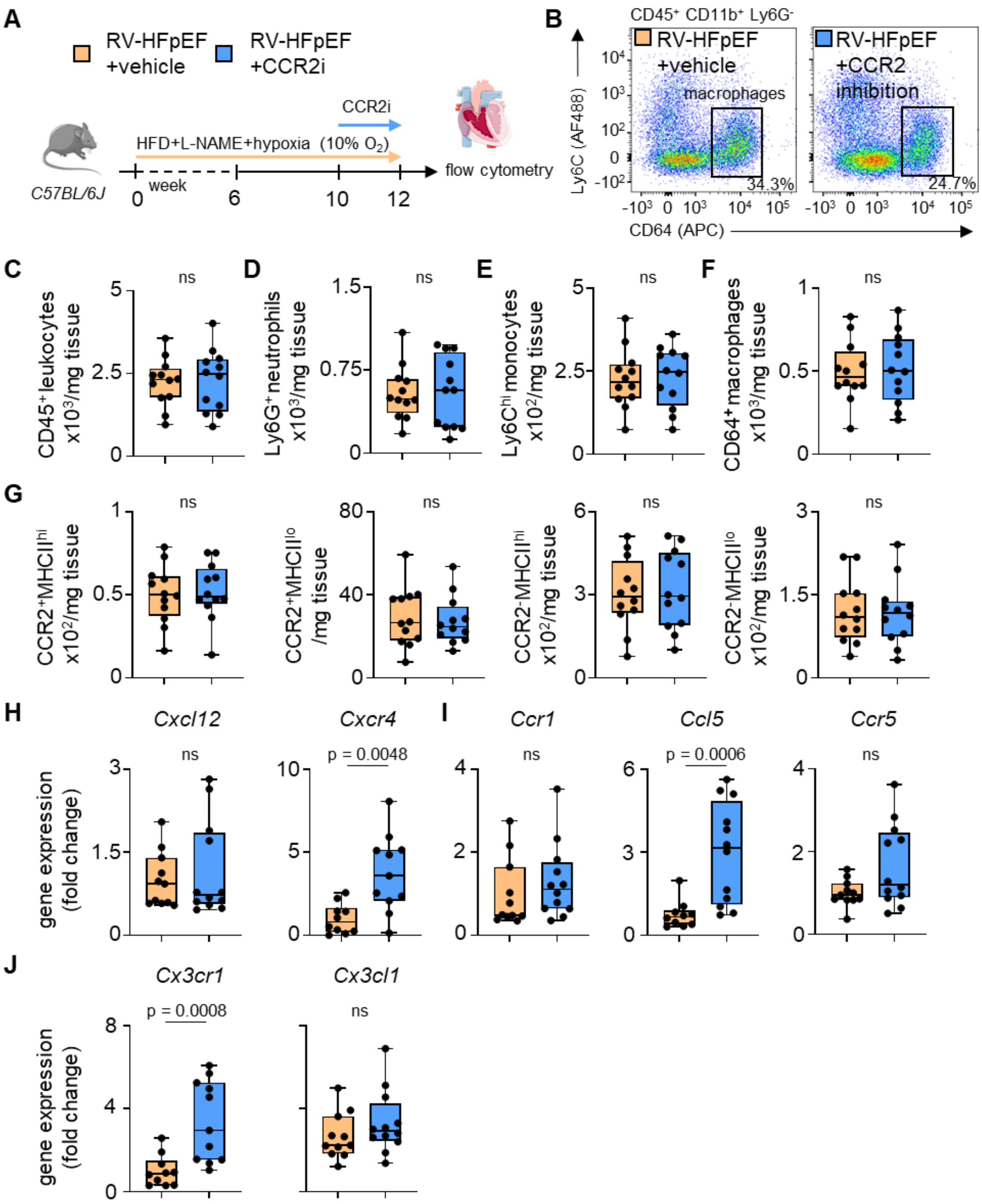

Figure S13 | Increased chemokine expression profiles may compensate effects of CCR2 inhibition in RV-HFpEF mice.

A, Experimental outline for RV-HFpEF mice treated with either vehicle (DMSO, RV-HFpEF+vehicle, 10 mg/kg/day) or CCR2 inhibitor (RS-102895, 10 mg/kg/day) for the last 2 weeks of the experiment (RV-HFpEF+CCR2i). **B**, Representative flow plots showing macrophages from RV-HFpEF+vehicle and RV-HFpEF+CCR2i mice. **C-G,** Flow cytometric quantification of $CD45^+$ leukocytes (**C**), $Ly6G^+$ neutrophils (**D**), $Ly6C^{hi}$ monocytes (**E**), $CD64^+$ macrophages (**F**) and $CCR2^+MHCII^{hi}$, $CCR2^+MHCII^{lo}$, $CCR2^-MHCII^{hi}$, $CCR2^-MHCII^{lo}$ macrophages (**G,** n=12). **H-J**, Quantitative PCR of *Cxcl12*, *Cxcr4* (**H**), *Ccr1*, *Ccl5*, *Ccr5* (**I**), *Cx3cr1 and Cx3cl1* (**J**) in RV-HFpEF+vehicle and RV-HFpEF+CCR2i RV bulk tissues (n=10-12). Expression values were calculated relative to *Sdha* expression using the $2^{-\Delta\Delta Ct}$ method. P values were calculated using a Mann–Whitney test (C-J). Data are presented as median with interquartile range (IQR). CCR2i, CCR2 inhibitor. HFpEF, heart failure with preserved ejection fraction. RV, right ventricle. HFD, high-fat diet. L-NAME, Nω-nitro-L-arginine methylester hydrochloride. ns, not significant.

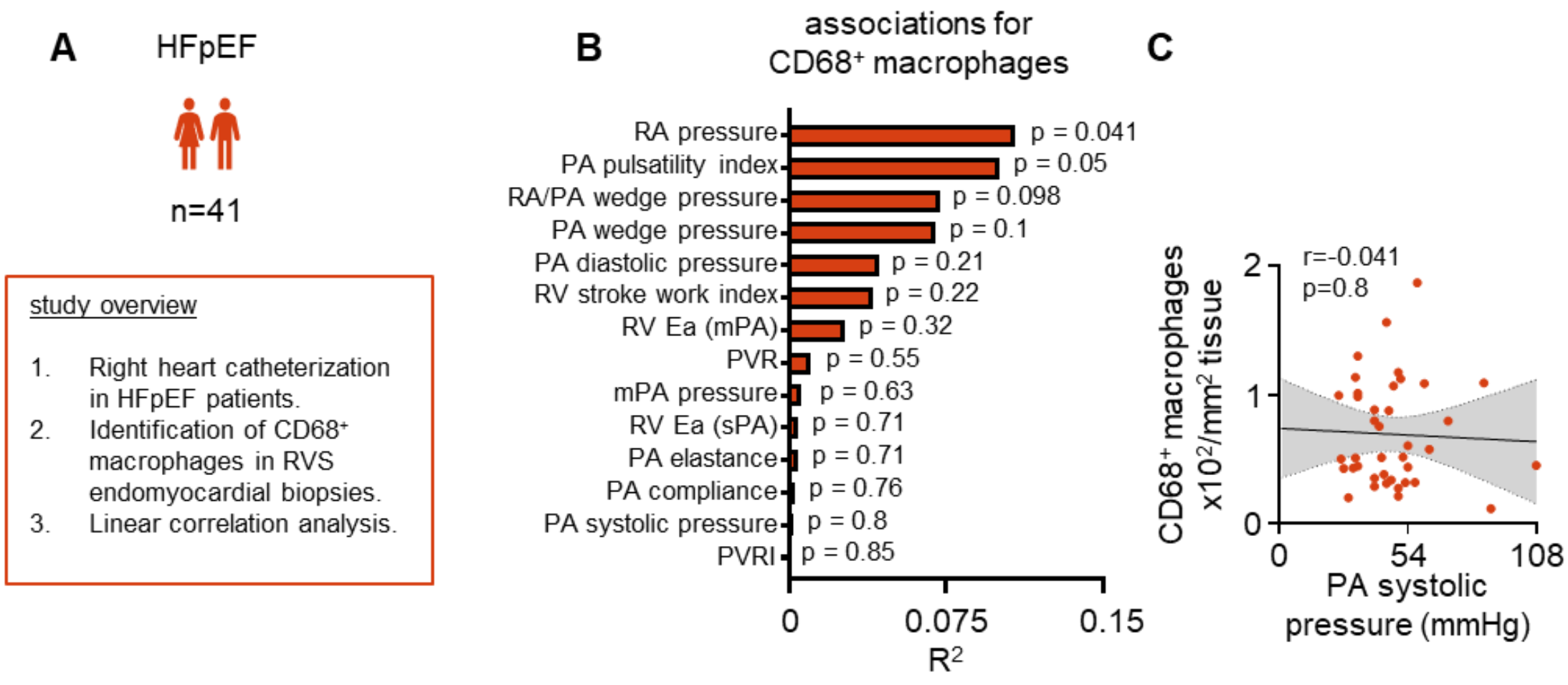

Figure S14 | CD68$^+$ cell counts are associated with clinical signs of RV dysfunction.
A, Study overview for the identification of CD68$^+$ macrophages in human HFpEF RVS biopsies. **B**, Linear correlation analyses using R^2 and 95% confidence interval reveal associations between clinical variables and CD68$^+$ cell counts from RVS biopsies in HFpEF patients. **C**, Linear correlation analysis with Pearson's r and 95% confidence interval between PA systolic pressure and CD68$^+$ cells. HFpEF, heart failure with preserved ejection fraction. RVS, RV septum. RV, right ventricle. PA, pulmonary artery. PVR, pulmonary vascular resistance. RA, right atrium.

Major resource table

Table 1 | Animals

Species	Age	Sex	Strain	Sources	Identifier
mice	8 weeks	Male and female	C57Bl/6J	Janvier Labs	https://janvier-labs.com/en/
mice	16-99 weeks	Male and female	C57Bl/6J	DIfE	n.a.
mice	8 week?	Male	C57Bl/6J	Charles River Laboratories	https://www.criver.com/products-services/research-models-services/animal-models/mice?region=23
mice	10-11 weeks	Male and female	$Cx3cr1^{CreER/+}$ $R26^{tdTomato/+}$	Institute of Microbiology, Infectious Diseases and Immunology FEM: 1253	n.a.

Table 2 | Anitibodies

Antibodies	Source	Clone	Identifier	Dilution
Anti-mouse CD45-BV711	BioLegend	30-F11	103147	1:1000
Anti-mouse CD11b-Pe-Cy7	BioLegend	M1/70	101216	1:1000
Anti-mouse CD3-Biotin	BioLegend	17A2	100244	1:300
Anti-mouse CD19-Biotin	BioLegend	6D5	115503	1:1000
Anti-mouse B220-Biotin	BioLegend	RA3–6B2	103204	1:100
Anti-mouse Nk1.1-Biotin	BioLegend	PK136	108704	1:100
Anti-mouse Ly6G-APC	BioLegend	1A8	127614	1:600
Anti-mouse CD115-BV605	BioLegend	AFS98	135517	1:600
Anti-mouse Streptavidin-APC-Cy7	BioLegend	n.a.	405208	1:250
Anti-mouse CD11b-APC-Cy7	BioLegend	M1/70	101226	1:600
Anti-mouse CD3-PE	BioLegend	17A2	100206	1:200
Anti-mouse CD19-PE	BioLegend	6D5	115507	1:300
Anti-mouse B220-PE	BioLegend	RA3–6B2	103207	1:300
Anti-mouse Nk1.1-PE	BioLegend	PK136	108707	1:300
Anti-mouse Ly6C-PerCP-Cy5.5	BioLegend	HK1.4	128012	1:600
Anti-mouse CD45-PerCP-Cy5.5	BioLegend	30-F11	103132	1:600
Anti-mouse CD11b-BV510	BioLegend	M1/70	101245	1:600
Anti-mouse Ly6C-AlexaFluor488	BioLegend	HK1.4	128006	1:600
Anti-mouse Ly6G-PE-Cy7	BioLegend	1A8	127617	1:600
Anti-mouse CD64-APC	BioLegend	X54–5/7.1	139305	1:600
Anti-mouse MHCII-APC-Cy7	Biolegend	M5/114.15.2	107628	1:600
Anti-mouse CCR2-BV605	BioLegend	SA203G11	150615	1:300
Anti-mouse CCR2-BV785	BioLegend	SA203G11	150621	1:200
Anti-mouse Ly6C-AF488	Biolegend	HK1.4	128021	1:600
Anti-mouse Ly6G-PE-Cy7	BioLegend	1A8	127618	1:600
Anti-mouse Ki67-PE	BioLegend	16A8	652403	1:300
Anti-mouse PE rat IgG2	BioLegend	RTK2758	400507	1:300
Anti-mouse TIMD4-BV786	BD Bioscience	RMT4-45	744631	1:400
Anti-mouse CCR2-RB780	BD Bioscience	475301	755695	1:400

Anti-mouse LYVE1-PE	Invitrogen	ALY7	12-0443-82	1:400
Anti-mouse Ly6G-PerCP-Cy5.5	BioLegend	1A8	127616	1:800
Anti-mouse NK1.1-PE-Cy5	BioLegend	PK136	108716	1:400
Anti-mouse CD19-Pe-Cy7	BioLegend	6D5	115519	1:1000
Anti-mouse CD3-R718	BD Bioscience	500A2	567303	1:100
Anti-mouse Ly6G	BioXcell	1A8	BP0075-1	n.a.
anti-rat Kappa Immunoglobulin Light Chain antibody	BioXcell	MAR	BE0122	n.a.
rat IgG2a	BioXcell	2A3	BP0089	n.a.
mouse IgG2a isotype control	BioXcell	C1.18.4	BE0085	n.a.

Table 3 | Softwares

Software	Version number	Source
FACSDiva	8.0.1	BD Bioscience
FlowJo	10.8.1	BD Bioscience
DirectDIA	Spectronaut version 19.5	Biognosys
R	4.4.3	n.a.
VevoLAB	5.7.1	FUJIFILM VisualSonics
VevoStrain	1	FUJIFILM VisualSonics
LabChart	8	ADInstruments
PhenoMaster	7.1.6	TSE Systems
NDP.scan SQ	1.0	Hamamatsu
GraphPad Prism	10	GraphPad

Table 4 | Drugs

Drug	Source	Identification	Validation
RS-102895	MedChemExpress	HY-18611A	HNMR, RP-HPLC, MS, SDS
PLX-5622	Hycultec GmbH	HY-114153	HNMR, LC-MS

Excel files

S1 | List of Reactome Pathways of Gene set enrichment analysis.

Reactome pathways and respective NES and FDR from RV-HFpEF over chow, RV-HFpEF over HFpEF, RV-HFpEF over Hx and RV-HFpEF RV over LV comparisons. NES, normalized enrichment score. HFpEF, heart failure with preserved ejection fraction, FDR, false dicovery rate. RV, right ventricle. LV, left ventricle. Hx, hypoxia

S2 | Protein abundance and intensities of proteomic analyses.

Differential abundance analysis results (logFC, t-statistic and FDR) of proteins in RV-HFpEF over chow, RV-HFpEF over HFpEF, RV-HFpEF over Hx and RV-HFpEF RV over LV comparisons and protein intensities measured in RV tissue of each individual animal of each group. HFpEF, heart failure with preserved ejection fraction, FDR, false dicovery rate. RV, right ventricle. LV, left ventricle. Hx, hypoxia
